# DNA methylation profiling identifies TBKBP1 as potent amplifier of cytotoxic activity in CMV-specific human CD8^+^ T cells

**DOI:** 10.1371/journal.ppat.1012581

**Published:** 2024-09-26

**Authors:** Zheng Yu, Varun Sasidharan-Nair, Thalea Buchta, Agnes Bonifacius, Fawad Khan, Beate Pietzsch, Hosein Ahmadi, Michael Beckstette, Jana Niemz, Philipp Hilgendorf, Philip Mausberg, Andreas Keller, Christine Falk, Dirk H. Busch, Kilian Schober, Luka Cicin-Sain, Fabian Müller, Melanie M. Brinkmann, Britta Eiz-Vesper, Stefan Floess, Jochen Huehn

**Affiliations:** 1 Department Experimental Immunology, Helmholtz Centre for Infection Research, Braunschweig, Germany; 2 Institute of Genetics, Technische Universität Braunschweig, Braunschweig, Germany; 3 Research Group Virology and Innate Immunity, Helmholtz Centre for Infection Research, Braunschweig, Germany; 4 Institute of Transfusion Medicine and Transplant Engineering, Hannover Medical School, Hannover, Germany; 5 German Center for Infection Research (DZIF), Thematical Translation Unit-Immunocompromised Host (TTU-IICH), partner site Hannover-Braunschweig, Germany; 6 Department Viral Immunology, Helmholtz Centre for Infection Research, Braunschweig, Germany; 7 Centre for Individualized Infection Medicine (CIIM), a joint venture of HZI and Hannover Medical School, Hannover, Germany; 8 Mikrobiologisches Institut–Klinische Mikrobiologie, Immunologie und Hygiene, Universitätsklinikum Erlangen und Friedrich-Alexander-Universität (FAU) Erlangen-Nürnberg, Erlangen, Germany; 9 Clinical Bioinformatics, Saarland University, Saarbrücken, Germany; 10 Helmholtz Institute for Pharmaceutical Research Saarland (HIPS)-Helmholtz Centre for Infection Research (HZI), Saarland University, Saarbrücken, Germany; 11 Institute of Transplant Immunology, Hannover Medical School, Hannover, Germany; 12 Institute for Medical Microbiology, Immunology and Hygiene, Technical University Munich (TUM), Munich, Germany; 13 German Center for Infection Research (DZIF), Thematical Translation Unit-Immunocompromised Host (TTU-IICH), partner site Munich, Germany; 14 FAU Profile Center Immunomedicine, Friedrich-Alexander-Universität (FAU) Erlangen-Nürnberg, Erlangen, Germany; 15 Integrative Cellular Biology and Bioinformatics, Saarland University, Saarbrücken, Germany; 16 Cluster of Excellence Resolving Infection Susceptibility (RESIST; EXC 2155), Hannover Medical School, Hannover, Germany; State University of New York Upstate Medical University, UNITED STATES OF AMERICA

## Abstract

Epigenetic mechanisms stabilize gene expression patterns during CD8^+^ T cell differentiation. Although adoptive transfer of virus-specific T cells is clinically applied to reduce the risk of virus infection or reactivation in immunocompromised individuals, the DNA methylation pattern of virus-specific CD8^+^ T cells is largely unknown. Hence, we here performed whole-genome bisulfite sequencing of cytomegalovirus-specific human CD8^+^ T cells and found that they display a unique DNA methylation pattern consisting of 79 differentially methylated regions (DMRs) when compared to memory CD8^+^ T cells. Among the top demethylated DMRs in cytomegalovirus-specific CD8^+^ T cells was *TBKBP1*, coding for TBK-binding protein 1 that can interact with TANK-binding kinase 1 (TBK1) and mediate pro-inflammatory responses in innate immune cells downstream of intracellular virus sensing. Since TBKBP1 has not yet been reported in T cells, we aimed to unravel its role in virus-specific CD8^+^ T cells. *TBKBP1* demethylation in terminal effector CD8^+^ T cells correlated with higher *TBKBP1* expression at both mRNA and protein level, independent of alternative splicing of *TBKBP1* transcripts. Notably, the distinct DNA methylation patterns in CD8^+^ T cell subsets was stable upon long-term *in vitro* culture. TBKBP1 overexpression resulted in enhanced TBK1 phosphorylation upon stimulation of CD8^+^ T cells and significantly improved their virus neutralization capacity. Collectively, our data demonstrate that TBKBP1 modulates virus-specific CD8^+^ T cell responses and could be exploited as therapeutic target to improve adoptive T cell therapies.

## Author summary

Human cytomegalovirus (CMV) is a herpesvirus that infects a significant portion of the global population. While it usually causes asymptomatic or mild infections, CMV can have severe consequences for individuals with a weakened immune system, such as stem cell or organ transplant recipients or individuals with HIV/AIDS. The immune response to CMV is characterized by expansion of virus-specific CD8^+^ T cells, which recognize and eliminate CMV-infected cells, thereby controlling the infection. Studies have shown that the pathogen-induced differentiation of naive to effector memory CD8^+^ T cells is accompanied by epigenetic changes. In order to identify the major genes involved in the functionality of CMV-specific CD8^+^ T cells, which are also regulated by DNA methylation, we compared the methylation profiles of CMV-specific CD8^+^ T cells with memory CD8^+^ T cells. As a result of this, we found that TBK-binding protein 1 (TBKBP1) plays a crucial role in the function of CMV-specific CD8^+^ T cells and enhances virus neutralization capacity upon overexpression, which has not been reported previously. This study opens the possibility of not only identifying unknown genes that contribute to the functionality of CMV-specific CD8^+^ T cells, but also potentially lead to improvements in adoptive T cell therapies.

## Introduction

The human cytomegalovirus (CMV) has a profound impact on the innate and adaptive immune system of the host during the three main infection phases: initial replication, persistence, and latency/reactivation [1]. A significant risk of non-relapse mortality has been reported in association with CMV reactivation after allogeneic hematopoietic stem cell transplantation (HSCT) [2,3]. Studies have shown that post-transplant CMV-infected patients have a low incidence of antiviral T cells and a delayed generation of virus-responsive T cells, suggesting that functional antiviral T cells are essential for eliminating viral infections [4,5]. During the initial period following HSCT, patients suffering from CMV reactivation exhibit a rapid reconstitution of their CD8^+^ T cell populations due to clonal expansion of CMV-specific T cells, leading to an unfavourable CD4:CD8 ratio [6–8]. Adoptive transfer of CMV-specific T cells has been shown to significantly reduce the risk of infection and reactivation of CMV in transplant recipients [9–13]. However, CMV-specific CD8^+^ T [T(CMV)] cells show a high degree of heterogeneity and possess distinct immunological functions [14–16]. Consequently, current adoptive transfer treatment strategies can still be improved to ensure optimal CD8^+^ T cell-mediated immunity.

Upon virus infection, naive CD8^+^ T cells are primed with viral antigen, undergo clonal expansion and differentiate into long-lived memory or terminally differentiated effector T cells [17,18]. During this differentiation process, CD8^+^ T cells acquire cytolytic functions that involve the action of multiple transcription factors like T-bet and Eomesodermin (EOMES), which co-operate to induce the expression of various effector molecules, including interferon gamma (IFN-γ), perforin, and granzyme B [19,20]. These effector CD8^+^ T cells then respond to their targets directly through antigen-specific cytotoxic activity and secretion of multiple cytokines and effector molecules. The human CD8^+^ T cell population consists of various functionally distinct subsets that can be distinguished through the changes in surface expression of homing markers and co-stimulatory molecules including CCR7, CD62L, CD27, CD28, and CD45RA during the differentiation process [21,22]. Consequently, these markers have been utilized to distinguish the phenotype of different human CD8^+^ T cell subsets, namely naive (T_N_), stem cell memory (T_SCM_), central memory (T_CM_), effector memory (T_EM_), and terminal effector memory T cells re-expressing CD45RA (T_EMRA_).

Accumulating evidence suggests that various epigenetic processes contribute to the T cell fate specification upon CD8^+^ T cell differentiation [23–25]. Changes in DNA methylation patterns and histone marks were shown to play a significant role in altering the transcriptional programme of effector- or stemness-related genes during differentiation of CD8^+^ T cell subsets [23,24,26]. Genome-wide profiling studies of naive and memory CD8^+^ T cell subsets revealed a dynamic distribution of histone marks, including H3K4me2, H3K4me3, and H3K27me3 [27–29]. In accordance with the assumption that DNA methylation in promoter regions is typically correlated with transcriptional inactivation [30,31], several studies have demonstrated that during CD8^+^ T cell differentiation a substantial loss of methylation marks in the promoter regions is followed by transcriptional activation of corresponding genes [32–34]. Although DNA methylation studies typically focus on promoter regions, the methylation processes observed in other genomic regions, including CpG islands/clusters (CGI), non-coding intergenic regions, and gene bodies also affect the transcription network [31]. Consequently, it is imperative to extend DNA methylation studies beyond classical promoter and transcriptional start site-specific analyses to gain a deeper understanding of the impact of epigenetic processes on T cell fate specification. Apparently, DNA methylation processes at distal regulatory domains, such as enhancers and CGIs, are often found to be negatively associated with both gene transcription and active histone modifications during cellular differentiation and phenotype commitment [35–37].

The current study focuses on the epigenetic characterisation of T(CMV) cells with respect to genome-wide DNA methylation patterns. We found that T(CMV) cells display a unique DNA methylation pattern consisting of 79 differentially methylated regions (DMRs) when compared to memory CD8^+^ T (T_mem_) cells. Among these epigenetic changes, we identified a DMR in the *TBKBP1* gene, which was further studied since its role for the cytotoxicity of CD8^+^ T cells is unknown. Intriguingly, *TBKBP1* was found to be highly expressed in T_EMRA_ cells at both mRNA and protein level, and its mRNA expression significantly correlated with the degree of demethylation. Although the *TBKBP1* DMR is closely located to an exon/intron junction, differences in DNA methylation did not result in distinct alternative splicing of the *TBKBP1* transcripts in CD8^+^ T cell subsets. Notably, the DNA methylation patterns of the *TBKBP1* DMR in T_N_ and T_EMRA_ cells remained stable upon long-term *in vitro* culture. Retroviral overexpression of TBKBP1 in CD8^+^ T cells resulted in TANK-binding kinase 1 (TBK1) activation, as evidenced by its increased phosphorylation status. Furthermore, CMV-specific T cells overexpressing TBKBP1 showed enhanced cytotoxicity against CMV-infected target cells along with augmented pro-inflammatory cytokine production. In summary, our results suggest that targeted demethylation of the *TBKBP1* DMR or overexpression of TBKBP1 in CD8^+^ T cells might improve the current adoptive T cell therapy for the prevention of CMV infection and relapse.

## Results

### T(CMV) cells exhibit a unique epigenetic signature

**Fig 1:**
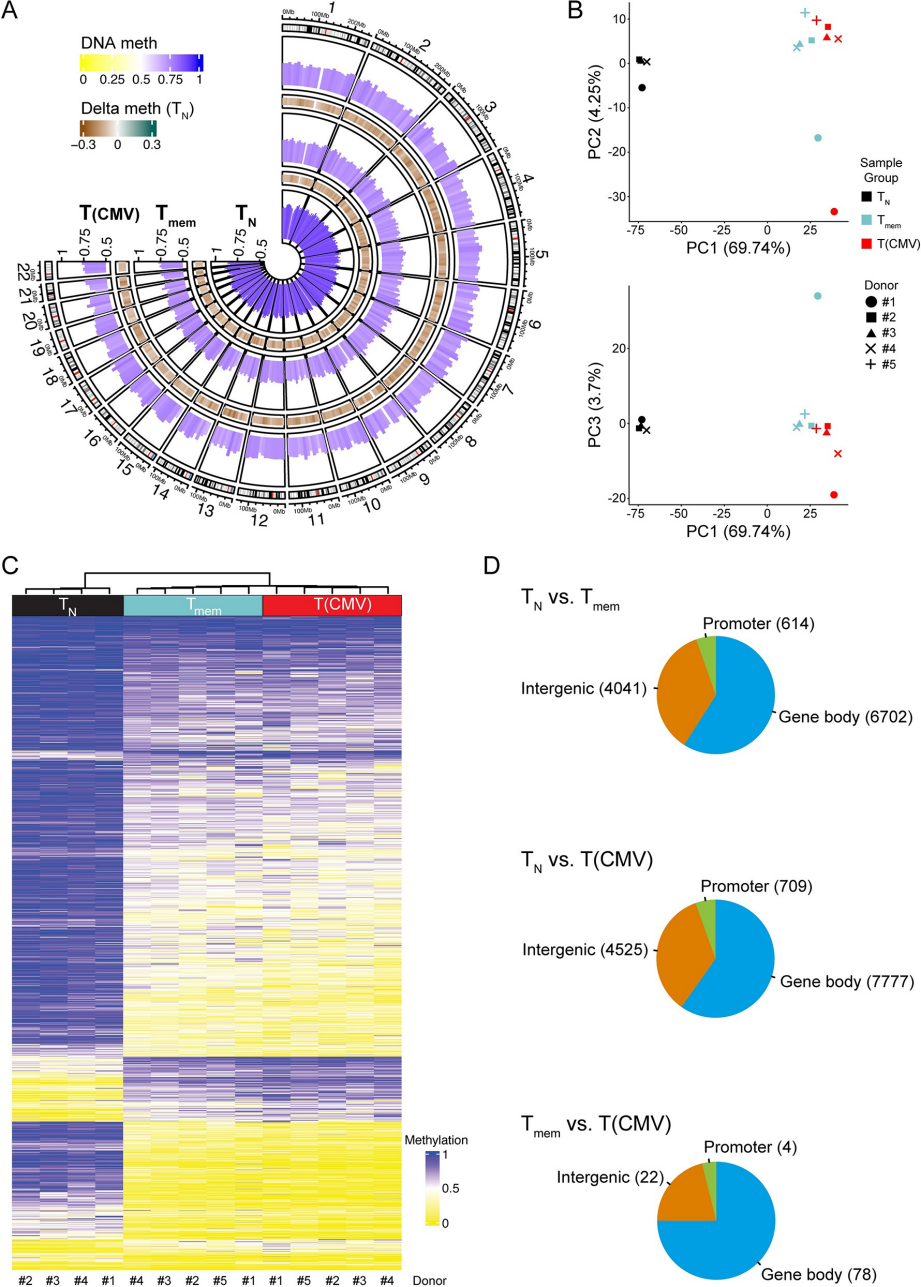
Genome-wide methylation profiling of T(CMV) cells. CD8^+^ T cell subsets including T_N_ (n = 4), T_mem_ (n = 5), and T(CMV) cells (n = 5) were sorted using flow cytometry. From each sample, genomic DNA was isolated and converted by bisulfite treatment, followed by WGBS. **(A)** Circos plot showing DNA methylation levels for T_N_, T_mem_, and T(CMV) cells across the whole genome (in 10 kb tiling windows, aggregated all donors). CpG methylation levels are represented as histogram tracks across the genome based on the sample. High levels of methylation are indicated by dark blue, while low levels of methylation are indicated by light yellow. Brown-teal colour-coding between the bar tracks indicates differences in methylation levels relative to T_N_ cells. **(B)** Principal Component Analysis (PCA) per cell type and donor samples based on 50,000 highly variable 1 kb tiling regions. The percent of explained variance for each component is denoted in the axis labels. **(C)** Hierarchical clustering of 15,598 non-overlapping DMRs identified in pair-wise comparisons of methylomes from T_N_, T_mem_, and T(CMV) cells. The dendrogram on top corresponds to hierarchical clustering of the samples. The colour-code illustrates the mean methylation levels of the DMRs as indicated (yellow: methylation level = 0%, blue: methylation level = 100%). **(D)** Pie charts indicating the position of the pairwise DMRs identified in indicated group-wise comparisons relative to annotated genes. Numbers in parentheses show the number of DMRs in intergenic, promoter, or gene body regions according to their genomic position.

The differentiation of naive murine CD8^+^ T cells into the different types of memory and effector T cells is associated with specific changes at the transcriptional and epigenetic level [23,24,29,38,39]. Studies on human CD8^+^ T cell memory formation similarly identified changes in DNA methylation patterns in the cytotoxicity-related genes *IFNG*, *GZMB*, and *PRF1* [40,41]. However, the global changes in DNA methylation patterns during the pathogen-induced formation of antigen-specific memory CD8^+^ T cells are only incompletely understood. Therefore, we isolated T(CMV) cells from 5 different CMV-seropositive donors to unravel their specific DNA methylation patterns. T(CMV) cells were identified as IFN-γ-secreting cells after restimulation with an overlapping peptide pool of the CMV-encoded pp65 protein (phosphoprotein of 65 kDa), which is the main component of the tegument layer of viral particles and an immunodominant target of T cell responses to CMV [42] (**S1A Fig**). An initial flow cytometric characterisation revealed that the vast majority of these IFN-γ^+^ T(CMV) cells showed a CD45RA^+^CD62L^−^ T_EMRA_ cell phenotype (**S1B Fig**), which is in line with recently published data [43,44]. For whole-genome bisulfite sequencing (WGBS), T(CMV) cells were isolated by flow cytometry-based cell sorting as CD3^+^CD4^−^CD8^+^IFN-γ^+^ cells, while memory CD8^+^ T cells (T_mem_; CD3^+^CD4^−^CD8^+^CCR7^−^CD28^high^CD27^+^CD45RA^−^; n = 5) and naive CD8^+^ T cells (T_N_; CD3^+^CD4^−^CD8^+^CCR7^+^CD28^int^KLRG1^−^CX3CR1^−^CD45RA^high^; n = 4) from the same donors served as controls (**S2 Fig**). For each sorted sample, genomic DNA was isolated and the genome-wide DNA methylation profile was determined by WGBS. Global analysis of the DNA methylation data revealed a strong progressive loss of DNA methylation from T_N_ to both T_mem_ and T(CMV) cells (**Fig 1A**), which is congruent with previously published data on human CD4^+^ memory T cells [45]. Next, we identified DMRs in pairwise comparisons of all T cell subsets. We found the highest numbers of DMRs between T_mem_ vs. T_N_ cells (11,357) and T(CMV) vs. T_N_ cells (13,011), while a rather low number of only 104 DMRs were identified in the comparison of T(CMV) vs. T_mem_ cells (**S1 Table**). Accordingly, a principal component analysis (PCA) based on genome-wide DNA methylation levels revealed a close sample group relationship between T(CMV) and T_mem_ cells, whereas T_N_ cells were placed separately along the main principal component 1 (PC1) (**Fig 1B**). This finding was confirmed by hierarchical clustering of individual samples, which further demonstrated that the majority of DMRs showed a consistent methylation status in T(CMV) and T_mem_ cells, while being distinct to the largely methylated state of most DMRs in T_N_ cells (**Fig 1C**). Yet, it is important to note that all T(CMV) and T_mem_ cell samples clustered group-wise, indicating distinct methylation signatures between these cell types. The majority of DMRs across all pair-wise population comparisons were located in gene bodies and intergenic regions, while a smaller fraction was mapped to promoters (**Fig 1D**). In line with previous findings [33,46], we observed that many genes involved in the CD8^+^ T cell effector program contain demethylated regions in both T(CMV) and T_mem_ cells, but are methylated in T_N_ cells, including genes coding for molecules regulating T cell-mediated cytotoxicity (*GZMA*, *GZMB*, *GZMK*, *IFNG*), actin cytoskeletal organization (*ACTA2*, *ACTB*, *ACTN4*, *DOCK2*), integrin-dependent cell adhesion (*ITGB1*, *ITGB1BP1*, *ITGA5*), chemokine-mediated signalling (*CCL1*, *CCL4*, *CCL5*, *CCR4*, *CCR5*), TGFβ-mediated signalling (*SMAD2*, *SMAD3*, *SMAD7*), and WNT/β-catenin signalling (*APC2*, *CTR9*, *CYLD*, *WNT11*, *WNT7B*) (**S1 Table**). Taken together, the WGBS study demonstrated substantial changes of DNA methylation patterns during the differentiation of T_N_ into T(CMV) cells.

**Fig 2:**
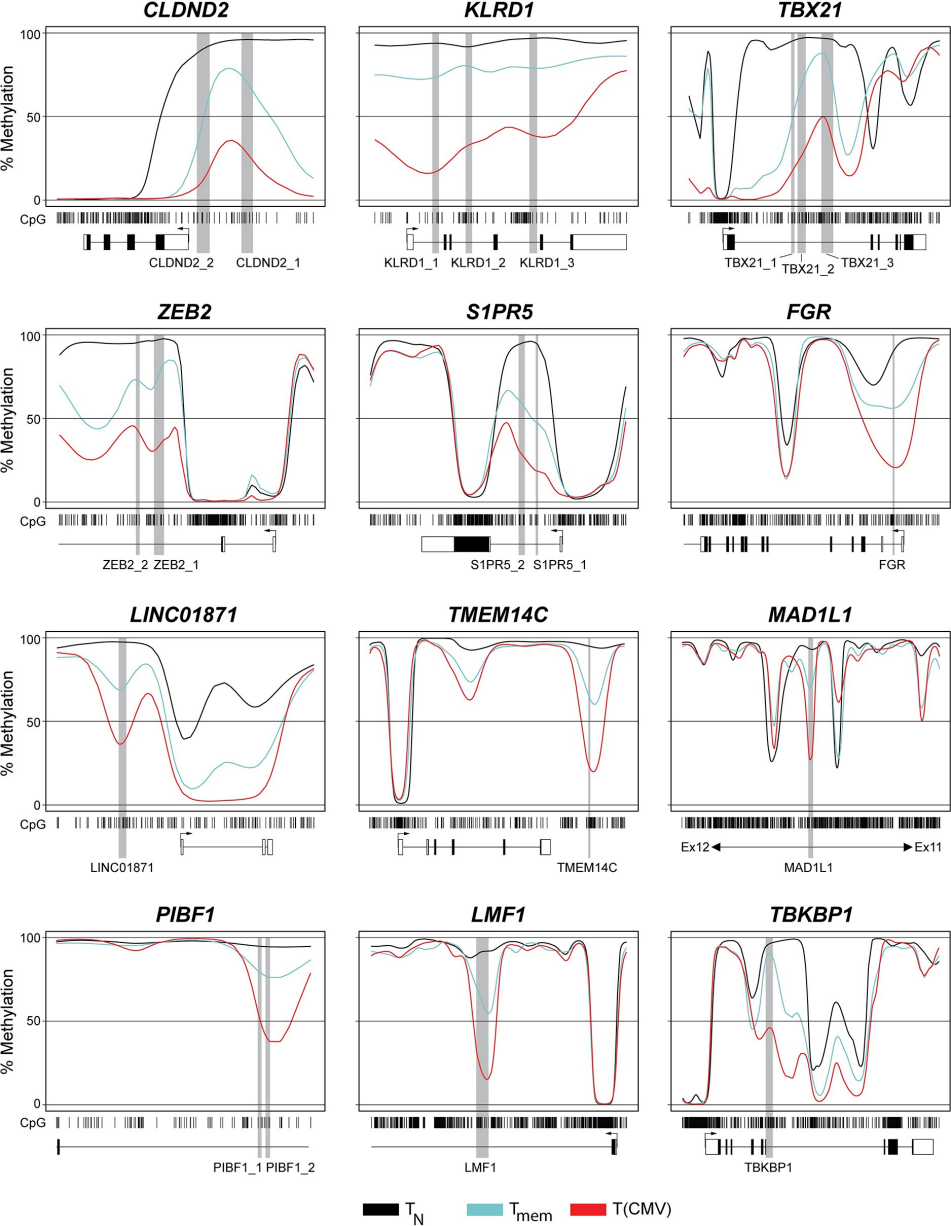
Methylation profiles of gene loci associated with top DMRs from epigenetic signature of T(CMV) cells. Out of the 71 genes showing unique differential DNA methylation patterns between T(CMV) and T_mem_ cells the top 12 DMR-associated gene loci were selected. For each gene, CpG motifs (barcodes), DMRs (light grey boxes), and exons of the surrounding gene body (dark grey boxes, transcriptional start site indicated by arrows) are displayed. Coloured lines illustrate methylation values ranging from 0–100% of T_N_ (black), T_mem_ (cyan), and T(CMV) cells (red) in a linear manner. Mean values from all donors per CD8^+^ T cell subset are depicted.

Next, we focused our analysis on the 104 DMRs that were identified in the comparison of T(CMV) vs. T_mem_ cells. A small fraction (25 DMRs) was also detected in the comparison of T_mem_ vs. T_N_ cells, and showed a more pronounced demethylation in T(CMV) cells (**S1 Table**). However, the majority (79 DMRs) was only detected in the comparison of T(CMV) vs. T_mem_ cells, and thus determine the unique epigenetic signature of T(CMV) cells. It is important to note that in a few cases more than one DMR was found in proximity to a given gene, resulting in 71 unique genes showing differential DNA methylation patterns between T(CMV) and T_mem_ cells (**S1 Table**). Some of the top demethylated DMRs in T(CMV) cells, identified in the pairwise comparison with T_mem_ cells, were associated to genes with known roles in differentiation and cytotoxic function of CD8^+^ T cells, including *KLRD1*, *TBX21*, *ZEB2*, and *S1PR5* [47–50] (**Fig 2**). In addition, a number of DMRs being selectively demethylated in T(CMV) cells were associated to genes for which a role in CD8^+^ T cells has not been described yet, namely *FGR*, *LINC01871*, *TMEM14C*, *MAD1L1*, *LMF1*, and *TBKBP1*. TBK-binding protein 1 (TBKBP1) is an adaptor protein that can interact with TBK1 and promote its activation [51,52]. TBK1 is a central player during the type I IFN and pro-inflammatory cytokine innate immune response by mediating phosphorylation and thereby activation of the transcription factors interferon regulatory factor (IRF) 3 and 7 and also NF-κB [53–59]. The transcription signature of CD8^+^ T_EMRA_ cells can be regulated by several innate immune-related genes, including natural killer (NK) cell activation mediators and multiple NK receptors [33]. These findings prompted us to study the role of TBKBP1 in CD8^+^ T cells in more detail.

### Demethylation of the *TBKBP1* DMR is associated with higher *TBKBP1* gene expression in effector memory T cell subsets but not with alternative splicing

**Fig 3:**
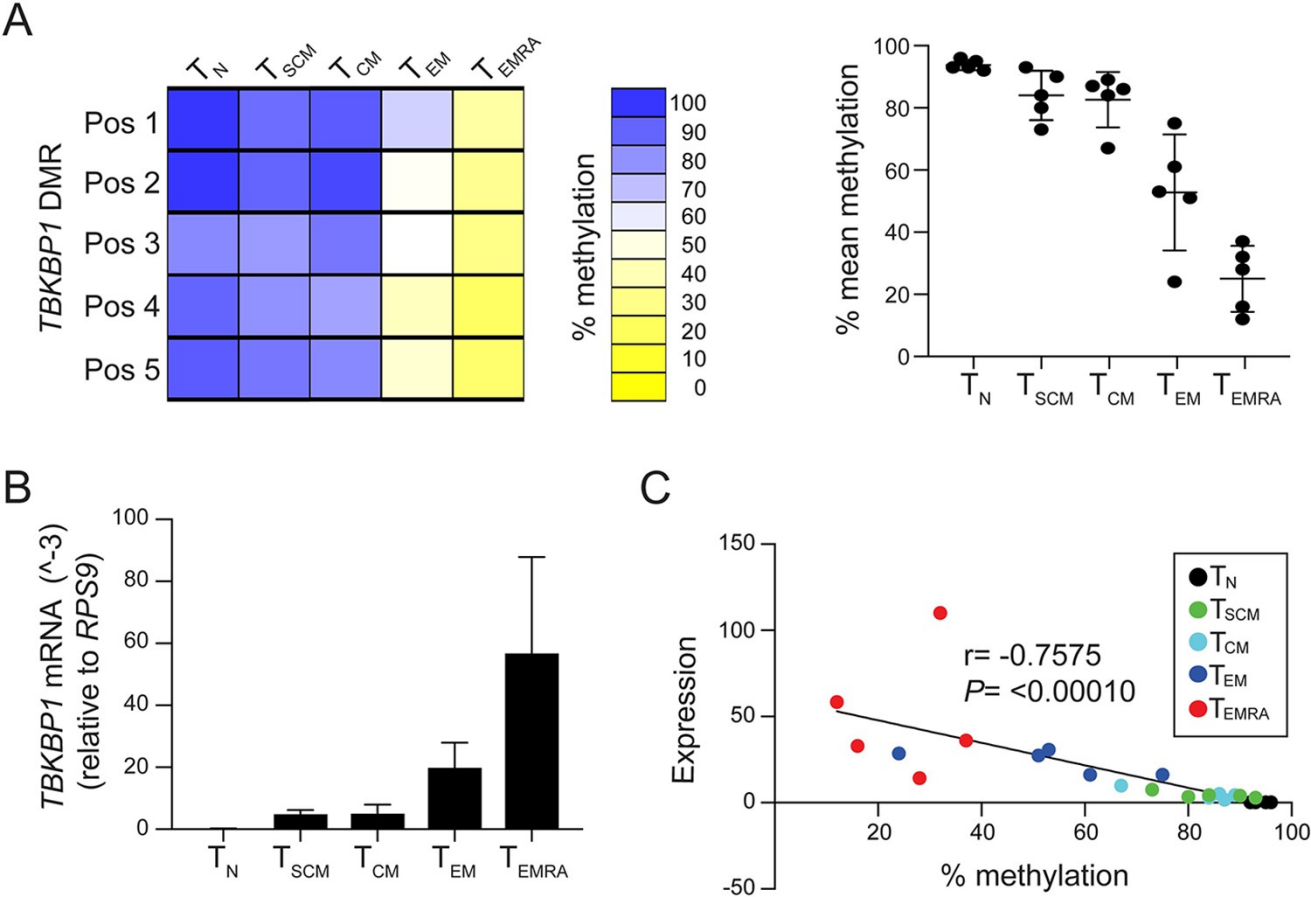
Demethylation of *TBKBP1* DMR correlates with increased *TBKBP1* expression in T_EMRA_ and T_EM_ CD8^+^ T cell subsets and is stably maintained upon *in vitro* culture. Indicated CD8^+^ T cell subsets were isolated from CMV-seropositive healthy donors and genomic DNA as well as RNA were isolated from sorted samples. Bisulfite-converted genomic DNA was subjected to pyrosequencing using primers targeting the *TBKBP1* DMR and RNA was transcribed into cDNA to determine *TBKBP1* expression levels by qRT-PCR. **(A)** Methylation profiles of the *TBKBP1* DMR in indicated CD8^+^ T cell subsets. (Left) The methylation values from 1 representative donor were translated into a colour-code according to the scale ranging from yellow (0% methylation) via white (50% methylation) to blue (100% methylation), the position of the CpG motifs is depicted, and each rectangle represents the methylation value of a single CpG motif. (Right) The scatter plot shows the mean methylation level of all 5 CpG motifs of the *TBKBP1* DMR in indicated CD8^+^ T cell subsets from 5 independent donors. Each dot represents data from one donor and mean values±SD are depicted. **(B)** The bar plot shows the relative *TBKBP1* expression normalized to the housekeeping gene *RPS9* in indicated CD8^+^ T cell subsets. Mean values±SD are depicted (n = 5). **(C)** Scatterplot and linear regression analysis show correlation of mean methylation of *TBKBP1* DMR with mean *TBKBP1* expression in indicated CD8^+^ T cell subsets from 5 donors (r, correlation coefficient; *p*, *p*-value).

To characterise the methylation status of the newly identified *TBKBP1* DMR within major human CD8^+^ T cell subpopulations, we isolated genomic DNA from sorted CD45RA^+^CCR7^+^ T_N_, CD45RA^+^CCR7^−^ T_EMRA_, CD45RA^−^CCR7^−^ T_EM_, CD45RA^−^CCR7^+^ T_CM_, and CD45RA^+^CCR7^+^CD28^+^CD62L^+^CD95^+^ T_SCM_ cells isolated from peripheral blood of CMV-seropositive donors and subjected them to pyrosequencing analysis. Analysis of the *TBKBP1* DMR showed a pronounced demethylation pattern in T_EMRA_ cells and a partial demethylation in T_EM_ cells, while T_CM_, T_SCM_ and particularly T_N_ cells were largely methylated at these sites (**Fig 3A**). Since it has been previously reported that CpG methylation levels around the promoter or first intron/exon region of a specific gene inversely correlate with gene expression [60–62], we next asked if the strong demethylation of the *TBKBP1* DMR in T_EMRA_ cells is accompanied by a high *TBKBP1* expression. Indeed, analysis of *TBKBP1* gene expression revealed a high expression in T_EMRA_ cells, an intermediate expression in T_EM_ cells, a low expression in T_CM_ and T_SCM_ cells, while *TBKBP1* expression was below the detection level in T_N_ cells (**Fig 3B**). Hence, we could observe a clear inverse correlation between DNA methylation rates and *TBKBP1* gene expression levels (r = -0.7575; *p*-value = <0.00010) (**Fig 3C**). Interestingly, we identified a nearly identical *TBKBP1* DMR methylation (**S3A Fig**) and mRNA expression pattern (**S3B Fig**) in sorted CD8^+^ T cell subsets isolated from human CMV-seronegative donors, also leading to an inverse correlation of the two data sets (r = -0.8965, *p*-value = <0.00010) (**S3C Fig**). In conclusion, our results indicate a DMR-mediated transcriptional control of the *TBKBP1* gene locus, which seems to be independent of the CMV status.

Besides altering the accessibility of DNA-binding transcription factors and thus the rate of transcription, changes in DNA methylation can also affect splicing, leading to the generation of alternative transcripts [63]. Since the *TBKBP1* DMR is located close to an exon/intron junction (**S4A Fig**), differences in DNA methylation at the *TBKBP1* DMR might result in distinct alternative splicing of the *TBKBP1* transcripts in CD8^+^ T cell subsets. Specific RT-PCRs were designed to discriminate between the different *TBKBP1* transcripts reported in the Ensembl genome browser (**S4A Fig**). However, the statistical analysis of the *TBKBP1-A*/*TBKBP1-B* transcript ratios of sorted T_CM_, T_EM_ and T_EMRA_ cells revealed no significant difference (**S4B Fig**), in line with a recently published study that did not detect TBKBP1 protein variants in total CD4^+^ and CD8^+^ T cells as well as NKT cells [64]. Thus, the DNA methylation changes at the *TBKBP1* DMR does not result in distinct alternative splicing of the *TBKBP1* transcripts.

### Stable *TBKBP1* DMR methylation pattern during long-term *in vitro* culture of CD8^+^ T cell subsets

**Fig 4:**
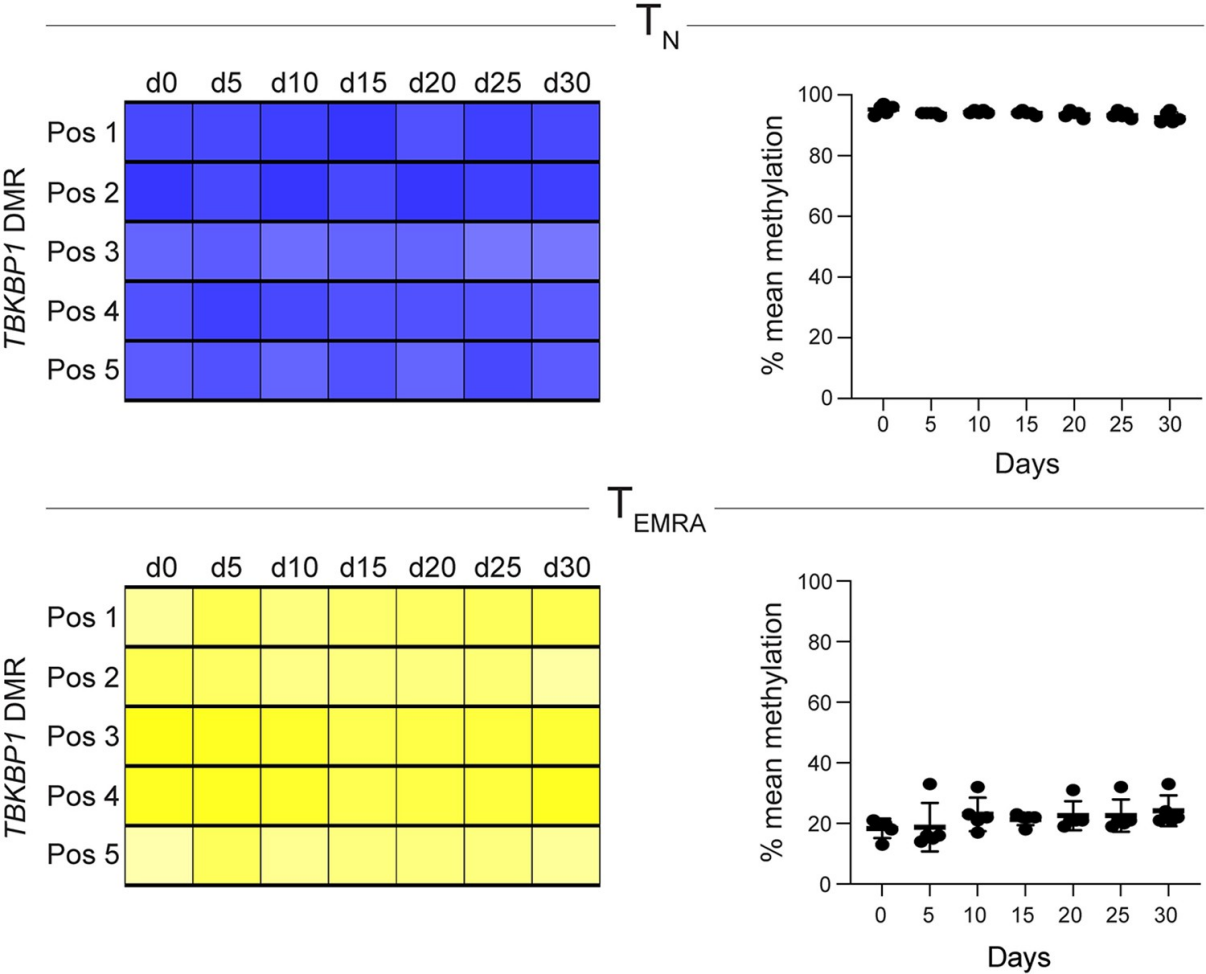
Long-term *in vitro* culture of CD8^+^ T cell subsets does not alter the *TBKBP1* DMR methylation patterns. CD8^+^ T_N_ and T_EMRA_ cells were isolated from CMV-seropositive healthy donors and cultured *in vitro* with plate-bound anti-CD3/CD28 antibodies in the presence of exogenous human IL-2 for up to 30 days. Every 5 days, samples from cultured T_N_ (top) and T_EMRA_ cells (bottom) were taken to analyse the *TBKBP1* DMR methylation status as described in Fig 3. The heatmaps are from a representative donor (left) and scatter plots summarize the mean methylation levels of all 5 CpG motifs of the *TBKBP1* DMR from 5 independent donors. Each dot represents data from one donor and mean values±SD are depicted.

During homeostatic proliferation, naive CD8^+^ T cells have been reported to undergo phenotypic alterations [65,66]. This observation raised the question whether the methylated and demethylated states of the *TBKBP1* DMR in T_N_ and T_EMRA_ cells, respectively, remain stable during proliferation. To answer this question, we isolated CD8^+^ T_N_ and T_EMRA_ cells from peripheral blood of CMV-seropositive healthy donors and cultured them for up to 30 days in the presence of anti-CD3 and anti-CD28 antibodies plus IL-2. Every 5 days, an aliquot was taken for phenotypic characterisation by flow cytometry and pyrosequencing. Upon culture of T_N_ cells, we observed a transient downregulation of the homing receptor CCR7 reaching normal levels by day 20, and an acquisition of stem-like properties with the upregulation of CD62L and CD28 on day 15 (**S5 Fig**), as recently reported [41]. In contrast, T_EMRA_ cells more stably maintained their CD45RA^+^CCR7^−^ phenotype throughout the culture. Pyrosequencing analyses revealed that the *TBKBP1* DMR remained fully methylated in cultured T_N_ cells and fully demethylated in cultured T_EMRA_ cells during the entire cultivation period (**Fig 4**). Together, these findings suggest that the epigenetic status of the *TBKBP1* gene is rather stable and preserved in both cultured T_N_ and T_EMRA_ cells even after multiple rounds of T cell receptor (TCR)-triggered cell division.

### Elevated TBKBP1 expression levels correlate with increased TBK1 phosphorylation in CD8^+^ T cells

**Fig 5:**
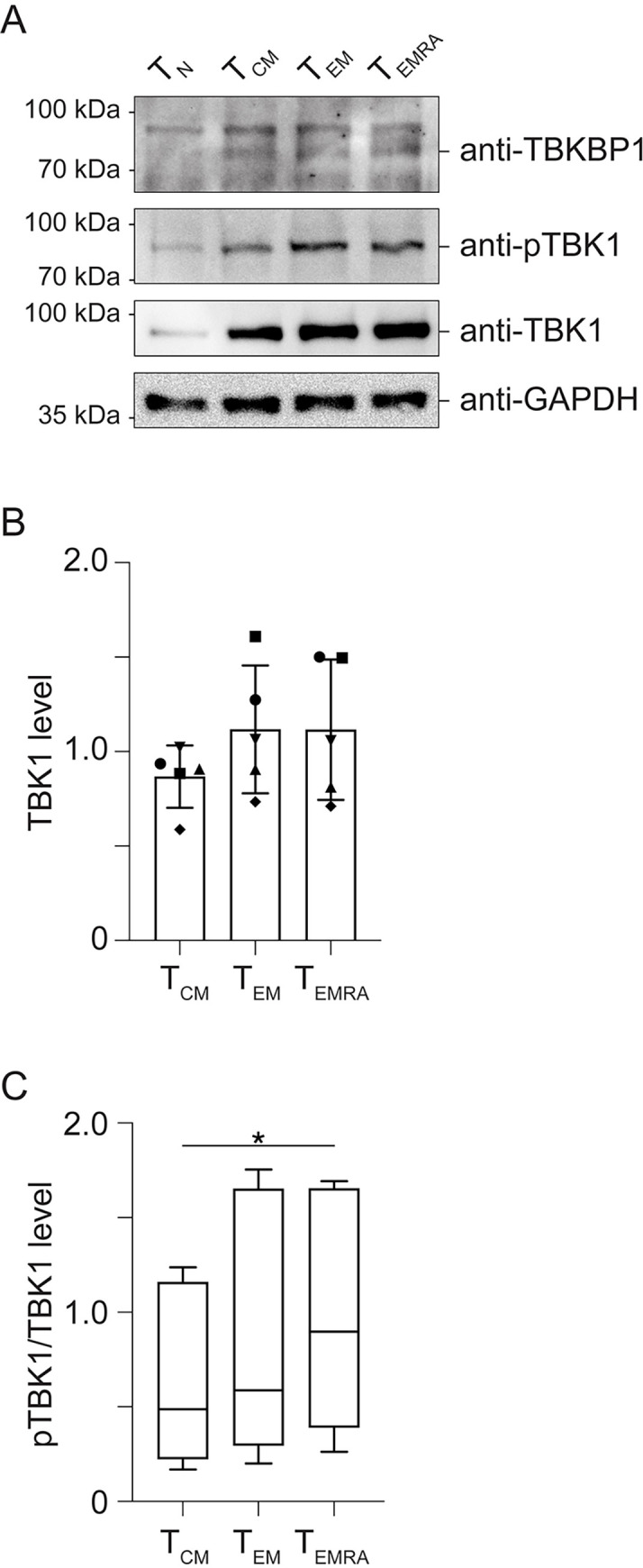
T_EM_ and T_EMRA_ cells display an increased phosphorylation of TBK1 when compared to T_CM_ cells. To characterize the status of the TBK1 pathway in CD8^+^ T cell subsets, T_N_, T_CM_, T_EM_ and T_EMRA_ cells were isolated from CMV-seropositive healthy donors, and lysates were generated and utilised for immunoblotting. **(A)** Representative immunoblot analysis of indicated CD8^+^ T cell subsets from one out of five donors showing the expression of TBKBP1, phosphorylated TBK1 (pTBK1) and TBK1. The analysis of GAPDH expression served as loading control. **(B)** The bar plots show GAPDH- or α-tubulin-normalized band intensities for TBK1 quantified from sorted T_CM_, T_EM_ and T_EMRA_ cells of 5 donors. Symbols indicate samples from the same donor. **(C)** The box-and-whiskers plots show the ratio of GAPDH- or α-tubulin-normalized band intensities of phosphorylated TBK1 and TBK1 from sorted T_CM_, T_EM_ and T_EMRA_ cells of 5 donors. For statistical analyses, a paired two-tailed student’s t test was conducted with *, p ≤ 0.05.

Although it is well known that the kinase TBK1 can be activated by various signals, including TCR signalling [53,67], the role of its adaptor protein TBKBP1 in CD8^+^ T cells remains unidentified. To characterize the status of the TBK1 signalling pathway in human CD8^+^ T cell subsets, we first analysed the total protein levels of TBKBP1 and TBK1 as well as phosphorylated TBK1 in T_N_, T_CM_, T_EM_, and T_EMRA_ cells *ex vivo* isolated from CMV-seropositive healthy donors by immunoblotting. In accordance with the *TBKBP1* mRNA expression data (**Fig 3B**), T_N_ cells hardly showed any TBKBP1 expression, while it was clearly detectable in T_CM_, T_EM_, and T_EMRA_ cells (**Fig 5A**). Similarly, TBK1 was only weakly expressed in T_N_ cells (**Fig 5A**), and T_CM_ cells showed slightly reduced TBK1 protein levels when compared to T_EM_ and T_EMRA_ cells, albeit not reaching statistical significance (**Fig 5B**). However, T_EMRA_ cells contained significantly more phosphorylated TBK1 when compared to T_CM_ cells (**Fig 5C**), indicating a higher basal activity of the TBK1 signalling pathway in terminally differentiated CD8^+^ T cells.

**Fig 6:**
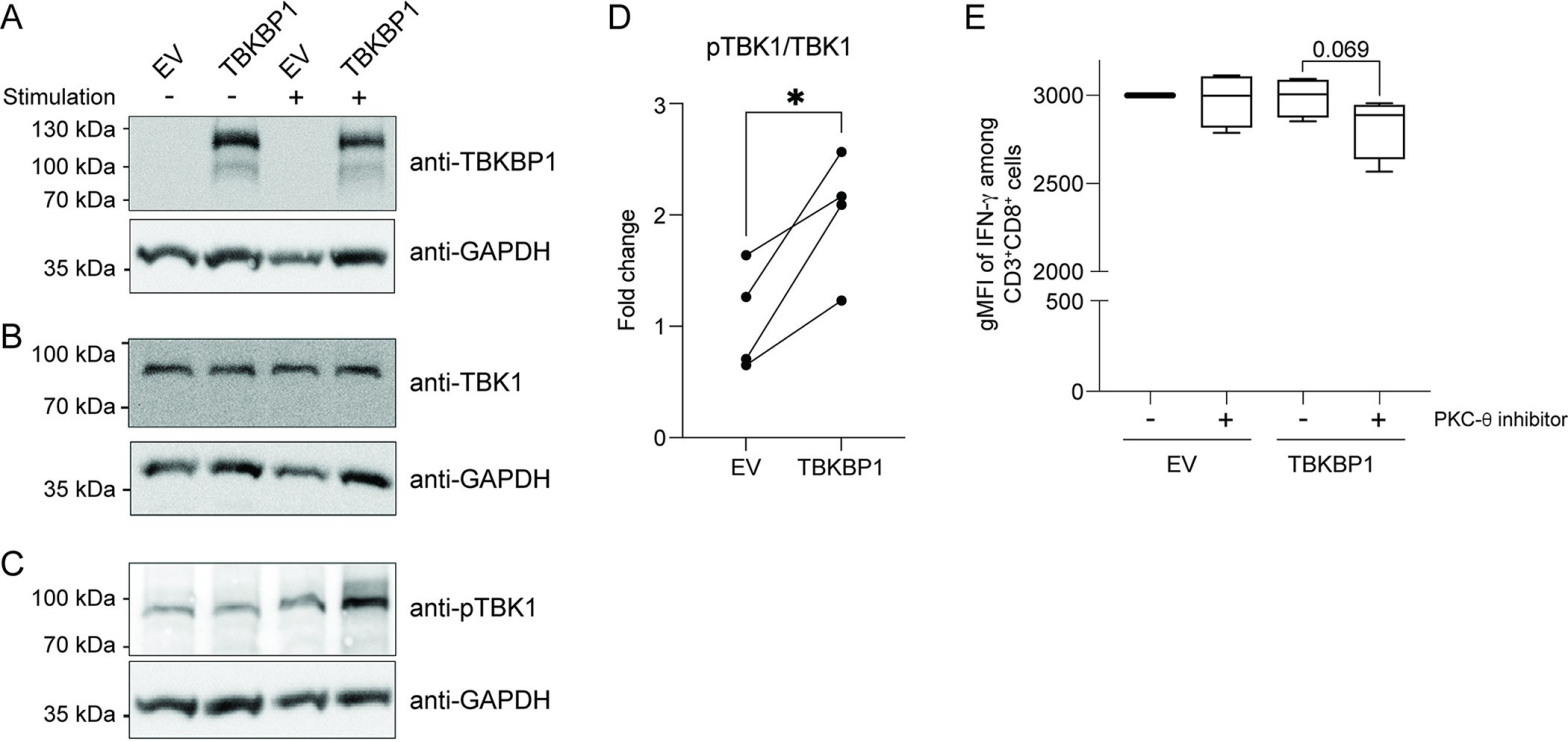
TBKBP1 overexpression in restimulated CD8^+^ T cells results in increased TBK1 phosphorylation. PBMCs obtained from CMV-seronegative healthy donors were stimulated with plate-bound anti-human CD3 and anti-human CD28 for 48 hours and subsequently retrovirally transduced with TBKBP1-expressing vectors or empty vector (EV) controls. Successfully transduced CD8^+^mCherry^+^ T cells were sorted by flow cytometry, serum-starved overnight, and subsequently stimulated with soluble anti-human CD3, anti-human CD28, and cross-linking antibodies, while unstimulated cells served as additional controls. Samples from both unstimulated (-) and stimulated (+) EV-transduced or TBKBP1-overexpressing CD8^+^ T cells were subjected to immunoblotting to determine the expression of **(A)** TBKBP1, **(B)** TBK1, and **(C)** phosphorylated TBK1 (pTBK1). The analysis of GAPDH expression served as loading control. **(D)** Band intensities for pTBK1 and TBK1 were quantified and the fold change of TBK1 phosphorylation upon stimulation of EV-transduced or TBKBP1-overexpressing CD8^+^ T cells was determined by dividing the ratio of pTBK1/TBK1 band intensities of stimulated cells to the ratio of pTBK1/TBK1 band intensities of unstimulated samples. Data are combined from 4 independent donors analysed in 2 independent experiments. **(E)** EV-transduced or TBKBP1-overexpressing CD8^+^ T cells were stimulated in the presence of a PKCθ inhibitor (+) or treated with DMSO as control (-). Subsequently, CD3^+^CD8^+^ cells were analysed for intracellular IFN-γ expression by flow cytometry. The geometric mean fluorescent intensity (gMFI) of IFN-γ expression among CD3^+^CD8^+^ cells is depicted. Data are combined from 4 independent donors analysed in 2 independent experiments. **(D, E)** For statistical analyses, a paired two-tailed student’s *t* test was conducted with *, p ≤ 0.05.

In a second step, we sought to get further insights into the role of TBKBP1 for the phosphorylation of TBK1. To this end, we retrovirally overexpressed TBKBP1 in CD8^+^ T cells and subsequently monitored TBK1 phosphorylation after short-term restimulation with anti-CD3 and anti-CD28 antibodies (**S6A Fig**). Successfully transduced CD8^+^ T cells were sorted by flow cytometry (**S6B Fig**) and TBKBP1-transdcued cells showed high *TBKBP1* mRNA expression levels when compared to EV-transduced controls as expected (**S6C Fig**). In addition, immunoblotting analysis confirmed high TBKBP1 protein expression in CD8^+^ T cells retrovirally transduced with the TBKBP1 overexpression construct, and no differences in total TBKBP1 expression were observed between unstimulated and short-term restimulated CD8^+^ T cells (**Fig 6A**). Similarly, neither TBKBP1 overexpression nor the short-term restimulation influenced total TBK1 expression in CD8^+^ T cells (**Fig 6B**). Notably, while EV-transduced control cells did not show any increase in TBK1 phosphorylation when short-term restimulated cells were compared to unstimulated controls, the phosphorylation of TBK1 was significantly enhanced in TBKBP1-overexpressing CD8^+^ T cells after short-term restimulation (**Fig 6C and 6D**). Since a recent report had demonstrated that TBKBP1 can support the recruitment of TBK1 to protein kinase C-theta (PKCθ) in murine lung epithelial cells, thereby promoting TBK1 phosphorylation and activation [52], we here also investigated the role of PKCθ for the TBK1 signalling pathway in CD8^+^ T cells. First, we assessed the impact of PKCθ on the phosphorylation of TBK1 by adding a highly selective PKCθ inhibitor (HY-112681) to EV-transduced or TBKBP1-overexpressing CD8^+^ T cells during short-term restimulation. As already mentioned above, immunoblotting analysis confirmed high TBKBP1 protein expression in TBKBP1-overexpressing CD8^+^ T cells, and the TBKBP1 overexpression did not influence total TBK1 expression (**S7 Fig**). Notably, upon addition of the PKCθ inhibitor we did not observe any effect on the phosphorylation of TBK1, neither in TBKBP1-overexpressing nor in EV-transduced control cells (**S7 Fig**), suggesting that PKCθ is not promoting the phosphorylation of TBK1 in CD8^+^ T cells under the tested stimulation conditions. However, the PKCθ inhibitor had an impact on the functional properties of TBKBP1-overexpressing CD8^+^ T cells and caused reduced IFN-γ expression levels after a 4-hour restimulation period when compared to DMSO-treated controls (**Fig 6E**). Interestingly, this inhibitory effect was only observed in TBKBP1-overexpressing CD8^+^ T cells and not in EV-transduced controls. Taken together, our findings suggest that TBKBP1 expression can promote TBK1 phosphorylation in activated CD8^+^ T cells in a PKCθ-independent manner, likely resulting in the activation of further downstream signalling pathways.

### Ectopic expression of TBKBP1 augments the virus-reducing capacity of CD8^+^ T cells

**Fig 7:**
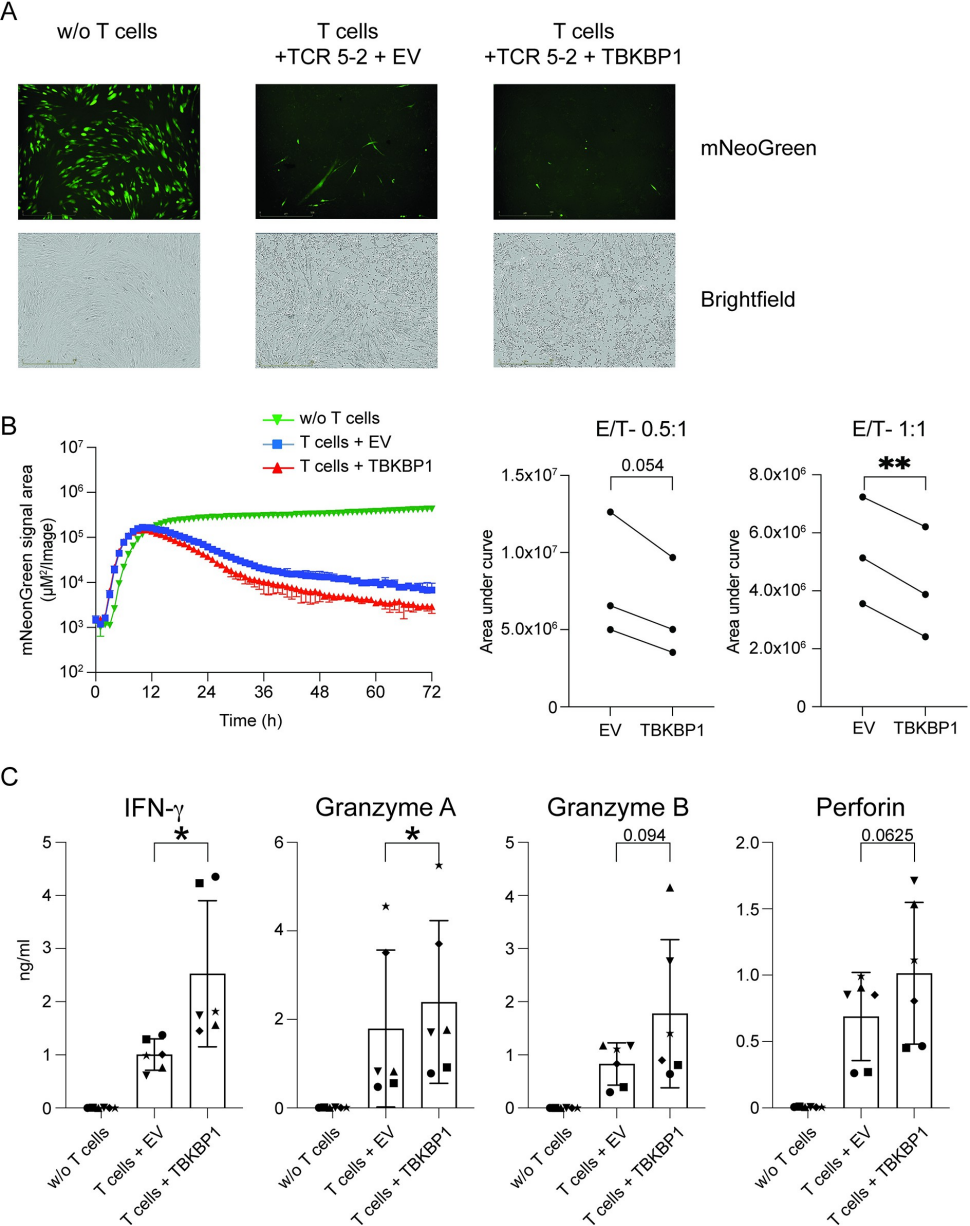
TBKBP1 overexpression enhances virus-reducing capacity of CD8^+^ T cells. PBMCs obtained from CMV-seronegative healthy donors were stimulated with plate-bound anti-human CD3 and anti-human CD28 for 48 hours and subsequently retrovirally transduced with both the high-avidity TCR 5–2 containing murine constant regions (mTCR) and directed against the CMV HLA-A*02-peptide NLVPMVATV and also TBKBP1-overexpressing vectors or empty vector (EV) controls. Successfully transduced mTCR 5–2^+^mCherry^+^ CD8^+^ T cells were sorted by flow cytometry and added at indicated effector:target (E:T) ratios to MRC 5 cells infected with recombinant CMV expressing the reporter protein mNeonGreen. ARMATA measures the expression of mNeonGreen using live cell imaging. **(A)** Representative microscopic images 48 hours after infection showing the expression of mNeonGreen (fluorescence image) and the location of the cells in corresponding brightfield images in CMV-infected MRC-5 cells in the absence of added T cells (w/o T cells), in the presence of EV-transduced mTCR 5–2^+^ CD8^+^ T cells (T cells + TCR 5–2 +EV) or in the presence of TBKBP1-overexpressing mTCR 5–2^+^ CD8^+^ T cells (T cells + TCR 5–2 +TBKBP1). T cells were added at a E:T ratio of 1:1. **(B)** The ARMATA was quantified by hourly measurements of the reporter signal in cultures in the absence of added T cells (w/o T cells, green curve), in the presence of EV-transduced mTCR 5–2^+^ CD8^+^ T cells (T cells + TCR 5–2 +EV, blue curve) or in the presence of TBKBP1-overexpressing mTCR 5–2^+^ CD8^+^ T cells (T cells + TCR 5–2 +TBKBP1, red curve). (Left) The graph depicts mean values±SD from 2 technical replicates of 1 representative out of 3 independent experiments. (Right) The line plots summarize data from 3 independent experiments by showing the average area under the curve (AUC) ±95% CI for EV-transduced or TBKBP1-overexpressing mTCR 5–2^+^ CD8^+^ T cells cultured with CMV-infected MRC-5 at indicated E:T ratios. **(C)** 36 hours after infection, culture supernatants were harvested and cytokine profiles were determined from cultures of CMV-infected MRC-5 cells in the absence of added T cells (w/o T cells), in the presence of EV-transduced mTCR 5–2^+^ CD8^+^ T cells (T cells + EV, E:T = 1:1) or in the presence of TBKBP1-overexpressing mTCR 5–2^+^ CD8^+^ T cells (T cells + TBKBP1, E:T = 1:1). Data from 3 independent experiments with 2 technical replicates each are shown. Symbols indicate samples from the same donor. For statistical analyses, a paired two-tailed student’s *t* test (leaving out “w/o T cell” group) was conducted with *, p ≤ 0.05 and **, p ≤ 0.01.

After having demonstrated that TBKBP1 overexpression has an effect on TBK1 phosphorylation, we next sought to investigate its functional role in CD8^+^ T cells during an immune reaction. To this end, we utilized ARMATA, an assay for rapid measurement of antiviral T cell activity [68]. In this assay, fibroblasts (MRC-5 cells) were infected with recombinant CMV expressing the fluorescent reporter mNeonGreen [69] and co-cultured with CMV-specific CD8^+^ T cells, which had been generated by retroviral overexpression of the human leukocyte antigen (HLA)-A*02:01-restricted high-avidity TCR recognising the CMVpp65-derived peptide NLVPMVATV (mTCR 5–2) [70]. Additionally, to assess the functional role of TBKBP1, cells were retrovirally transduced with the TBKBP1 overexpression construct, while EV-transduced cells served as control. Successfully transduced mTCR 5–2^+^mCherry^+^ CD8^+^ T cells were sorted by flow cytometry (**S8 Fig**) and added to the CMV-infected MRC-5 cells at different effector:target (E:T) ratios. The ARMATA measures the viral expression of mNeonGreen by temporal live cell imaging in 1-hour intervals and capture of representative microscopic images. As shown in **Fig 7A**, we observed a strong antiviral and cytopathic effect of mTCR 5–2^+^ CD8^+^ T cells, with an overall reduced number of viable mNeonGreen-expressing MRC-5 cells when compared to controls lacking T cells. Hourly measurements of the reporter signal revealed a continuous increase of the reporter signal in cultures without T cells, reaching a plateau around 12 hours with an only mild increase afterwards (**Fig 7B**, green curve). Upon addition of EV-transduced mTCR 5–2^+^ control CD8^+^ T cells, a marked decrease of the reporter signal was observed, starting at 12 hours and continuing until the end of the assay at 72 hours (**Fig 7B**, blue curve). This signal reduction was even more pronounced when TBKBP1-overexpressing mTCR 5–2^+^ CD8^+^ T cells were added, showing a significantly stronger neutralization of MRC-5-infected cells irrespective of the E:T ratio (**Fig 7B**, red curve). The improved virus reduction by TBKBP1-overexpressing CD8^+^ T cells was associated with significantly elevated levels of IFN-γ and Granzyme A, and a trend towards increased levels of Granzyme B, Perforin, MIP-1α (CCL3), and MIP-1β (CCL4) in supernatants of co-cultures with TBKBP1-overexpressing CD8^+^ T cells when compared to EV-transduced controls (**Figs 7C and S9**). Collectively, these data demonstrate that ectopic expression of TBKBP1 in human CD8^+^ T cells results in the upregulation of pro-inflammatory cytokines and chemokines, facilitating a significantly enhanced virus neutralization capacity.

## Discussion

Epigenetic processes, including DNA methylation, are well-known contributors to T cell fate specification upon CD8^+^ T cell differentiation [23–25]. In the present study, we hypothesized that global DNA methylation analyses could provide insights into the epigenetic reprogramming of T(CMV) cells and also support the identification of key players involved in their effector functions. Thus, we compared the DNA methylation profile of CMVpp65-reactive T(CMV) cells with the DNA methylation profile of antigen-experienced CD8^+^ T cells (CCR7^−^CD28^high^CD27^+^CD45RA^−^) with unknown specificity. Utilizing WGBS, we observed substantial epigenetic alterations upon T cell differentiation, and the vast majority of identified DMRs were demethylated in both T_mem_ and T(CMV) when compared to T_N_ cells. With the final aim to identify an epigenetic signature of T(CMV) cells, we focused our analysis on the comparison of DNA methylation profiles of T_mem_ and T(CMV) cells, and could identify 79 regions being differentially methylated between T(CMV) and T_mem_ cells and not between T_mem_ and T_N_ cells, thus representing a unique epigenetic signature of T(CMV) cells. One of these epigenetic signature genes was *TBKBP1*, not described in the context of T cell-mediated immune responses so far. The identified *TBKBP1* DMR was found upstream of exon 6, and it is tempting to speculate that transcription factors binding to this DMR might co-operate with the known *TBKBP1* enhancer region located between exon 4 and 5 (Ensembl ENSR00001010382).

The pronounced demethylation of the *TBKBP1* DMR in T(CMV) cells encouraged us to investigate the specific role of TBKBP1 for the cytotoxic function of CD8^+^ T cells. We could validate the preferential demethylation of the *TBKBP1* DMR in T_EMRA_ cells, the dominant phenotype of T(CMV) cells, which also showed the highest *TBKBP1* mRNA expression levels and the highest levels of phosphorylated TBK1, suggesting a higher basal activity of the TBK1 signalling pathway in terminally differentiated CD8^+^ T cells. Yet, the demethylation of the *TBKBP1* DMR, and probably the increased activity of the TBK1 signalling pathway, does not seem to be restricted to CMV-specific T cells, since we also found a pronounced *TBKBP1* DMR demethylation correlating with enhanced *TBKBP1* mRNA expression in T_EMRA_ cells from CMV-seronegative donors. Since other viruses, such as dengue, are known to promote the formation of T_EMRA_ cells [71], our findings suggest that the observed epigenetic regulation of *TBKBP1* expression could be also caused by immune responses to other pathogens.

A direct involvement of TBKBP1 in the phosphorylation of TBK1 was confirmed using TBKBP1-overexpressing cells, which exhibited a significantly increased TBK1 phosphorylation upon TCR triggering. This suggests that TBKBP1 is crucial for the optimal TCR-induced activation of TBK1 and downstream signalling pathways. Accordingly, TBKBP1 overexpression resulted in an increased production of pro-inflammatory cytokines and chemokines and promoted the cytotoxicity of T(CMV) cells against CMV-infected target cells. Collectively, these observations demonstrate the crucial role of TBKBP1 for optimal adaptive T cell responses and contribute to our current understanding of CD8^+^ T cell responses to CMV. However, it must be noted that overexpression systems might cause aberrant effects when compared to endogenous expression of a given protein. Thus, to further understand the role of TBKBP1 for the TBK1 signalling pathway and antiviral mechanisms in CD8^+^ T cells, the impact of endogenous TBKBP1 expression requires further investigation.

The role of DNA methylation for T cell fate specification has mostly been studied in relation to cytokines and effector molecules [41,72,73]. More recently, a number of genome-wide epigenome profiling studies gave first insights into the global changes upon T cell differentiation [23,25,27,28,35,36,45]. A seminal comparative study on acute and chronic viral infections demonstrated that naive antigen-specific CD8^+^ T cells are epigenetically regulated and differentiate rapidly into either a memory or exhaustive phenotype upon infection [73]. Interestingly, a recent report showed that even during their early phase of differentiation, CD8^+^ T cells responding to acute and chronic infections displayed distinct transcriptional and epigenetic landscapes [74]. Among the unique epigenetic signature genes of T(CMV) cells identified in the present study, *KLRD1*, *TBX21* (coding for T-bet), and *ZEB2* have already been reported to regulate functional properties of memory CD8^+^ T cells and were found to be upregulated in chronic infections or exhausted CD8^+^ T cells [14,49,74–78]. Another gene locus, *ADAM28*, which was recently identified in an ATACseq-based epigenetic profiling study comparing human T_EMRA_ with T_CM_/T_EM_ cells [79], was not among the unique epigenetic signature genes of T(CMV) cells, but only found when T(CMV) were compared to T_N_ cells. These findings suggest that small differences in sorting strategies to isolate CD8^+^ T cell subsets or the use of different epigenetic profiling methods might result in different unique epigenetic signature genes.

Furthermore, we noted that DMRs linked with *S1PR5*, a migratory receptor that is expressed by circulating memory CD8^+^ T cells, but selectively downregulated in tissue-resident memory CD8^+^ T cells [50,80,81], were demethylated in T(CMV) cells. A recent report on murine tissue-resident memory T cells demonstrated that *S1pr5* induction is directly controlled by T-bet and Zeb2 [50]. Additionally, CD8^+^ T cells from chronic infection models of lymphocytic choriomeningitis virus exhibit a higher frequency of T-bet^+^ cells and higher T-bet expression levels than those from acute infection models [74]. These reports further strengthen the findings from our DNA methylation dataset and suggest that in T(CMV) cells, the selectively demethylated genes *TBX21*, *ZEB2*, and *S1PR5* may jointly coordinate the effector function of antigen-specific CD8^+^ T cells.

Moreover, we identified a DMR associated with *TBKBP1* to be strongly demethylated in T(CMV) cells. TBKBP1 was initially reported to be engaged in the TNFα/NF-κB pathway to elicit innate immune responses [51]. The antiviral response of the innate immune system relies on various inducible transcription factors such as NF-κB and IRFs, which account for the production of IFNs and pro-inflammatory cytokines [82–84]. Studies showed that IκB kinase-I (IKKε), TNFR-associated factor (TRAF)-dependent NF-κB activator, and TBK1 trigger the phosphorylation of IRF3 and IRF7, which are responsible for transactivating type I and type II IFNs [54,55]. A recent report demonstrated that TBK1 can be recruited by TBKBP1 to interact with protein kinase C-theta (PKCθ), thereby enabling TBK1 phosphorylation and activation [52]. Yet, using a highly selective PKCθ inhibitor, we could not observe any impact of PKCθ on the phosphorylation of TBK1. However, the findings from the overexpression approach support a role of TBKBP1 in CD8^+^ T cells, which upon restimulation showed an enhanced TBK1 phosphorylation that could potentially support downstream signalling pathways. Moreover, various reports showed that phosphorylation of multiple serine residues within regulatory regions of IRF3 and IRF7 orchestrates TBK1 and IκB-mediated signalling pathways [85,86], that IRF7 can promote the expression of IFN-γ-responsive genes through the initiation and stabilization of RNA polymerase II recruitment in the presence of TBK1 and/or IKKε [85], and that IRF3 affects granzyme B expression and maintenance of memory T cell function in response to viral infection [87]. These findings are in line with data from the present study demonstrating that ectopic expression of TBKBP1 enhances the cytotoxic activity of CD8^+^ T cells through the upregulation of several pro-inflammatory mediators such as IFN-γ, granzyme A, granzyme B, and perforin. Collectively, our data indicate that TBKBP1 promotes the activation of TBK1 and further downstream pathways for optimal effector function of CMV-specific CD8^+^ T cells.

In conclusion, our findings provide evidence for the potential utility of TBKBP1 as a therapeutic target for the modulation of CD8^+^ T cell-mediated immune responses. Clinical relevance of our findings lies in the fact that ectopic expression of TBKBP1 enhanced the cytotoxicity of T(CMV) cells, suggesting that TBKBP1 could be exploited as a potential therapeutic target for the improvement of adoptive T cell therapy in CMV-infected individuals.

## Material and methods

### Ethics statement

All human samples were obtained from the Institute of Transfusion Medicine and Transplant Engineering, Hannover Medical School (MHH). Written informed consent was obtained from all donors (Ethics Committee MHH, approval numbers: 3639–2017, 9001_BO-K, 9255_BO_K_2020).

### Human donors

All human samples were obtained from male healthy donors. For the phenotypic characterisation and isolation of T(CMV) cells, CMV-seropositive HLA-typed donors showing >0.5% CMV phosphoprotein 65 (pp65)-specific T cells upon antigen-specific restimulation [88,89] were selected. The PKCθ inhibition experiments and the ARMATA were performed using cells from male CMV-seronegative, HLA-A*02 negative donors. *In vitro* cultivation experiments were conducted using cells from male CMV-seropositive donors regardless of their HLA type. All remaining experiments were performed with samples from both CMV-seronegative and -seropositive donors regardless of their HLA type. CMV-serological testing and HLA-typing were conducted at the Institute of Transfusion Medicine and Transplant Engineering, MHH.

### Human sampling and cell isolation

For the phenotypic characterisation and whole genome bisulfite sequencing (WGBS) of T(CMV) cells, PBMCs were isolated from residual blood samples from disposable kits used during routine platelet apheresis at the Institute of Transfusion Medicine and Transplant Engineering, MHH, via discontinuous gradient centrifugation and immediately processed. For all remaining experiments, PBMCs were isolated from Leukocyte Reduction System (LRS) cones using Ficoll-based density gradient centrifugation (Lymphoprep; STEMCELL Technologies) and SepMate tubes (STEMCELL technologies). PBMCs were cryopreserved in FCS containing 10% DMSO. One day prior to the experiment, cryopreserved PBMCs were thawed, rinsed with excess TexMACS medium (Miltenyi Biotec) to remove DMSO, and rested overnight with TexMACS medium supplemented with 100 IU/ml of recombinant human IL-2 (Miltenyi Biotec).

### Antibodies for flow cytometry

All monoclonal antibodies used for immunophenotyping and cell sorting were purchased from BioLegend and BD Biosciences. Characterisation of T(CMV) cells was performed using anti-CD3 BV510, anti-CD8 FITC, anti-IFN-γ PE, anti-CD45RA PerCP/Cy5.5, and anti-CD62L AF647. For WGBS, T(CMV) cells and control T cell subsets were isolated using anti-CD3 AF488, anti-CD4 APC-Cy7, anti-CD8 APC, anti-CD27 BV510, anti-CD28 PE-Dazzle, anti-CD45RA PerCP/Cy5.5, anti-KLRG1 PE/Cy7, anti-CCR7 BV421, and anti-CX3CR1 BV650. For pyrosequencing, quantitative real-time-PCR (qRT-PCR), and *in vitro* cultivation experiments, CD8^+^ T cell subsets were isolated using anti-CD3 AF488, anti-CD8 APC, anti-CD28 PE Texas Red, anti-CD45RA PerCP/Cy5.5, anti-CD62L PE-Cy7, anti-CD95 PE, and anti-CCR7 BV421. For the isolation of retrovirally-transduced CMV-specific CD8^+^ T cells, anti-CD8 APC and anti-mouse TCR APC-Cy7 along with LIVE/DEAD Fixable Blue Dead Cell dye were used (ThermoFischer Scientific).

### Flow cytometry

PBMCs were prepared for flow cytometry assay as described previously [90]. Briefly, for surface staining, cells were resuspended in staining buffer (PBS and 0.5% BSA), and single-cell suspensions were labelled with antibodies for 30 minutes at 4°C, while chemokine receptor staining was carried out at 37°C for 30 minutes. For intracellular staining of IFN-γ, the Foxp3 staining kit (eBioscience) was used according to the manufacturer’s protocol. RatIgG (Dianova, final concentration of 40 μg/ml) was added to block unspecific binding. Following staining, cells were washed and resuspended in staining buffer. Samples were acquired on a FACSCanto10c, LSR-II flow cytometer (BD Biosciences) or LSRFortessa flow cytometer (BD Biosciences). Data were analysed using BD FACSDiva software version 8.0.1 and FlowJo software version 10 (both from BD Biosciences). Cell sorting of CD8^+^ T cell subsets and transduced cells was performed on a BD FACS ARIA II SORP (BD Biosciences).

### Phenotypic characterisation of T(CMV) cells

For the phenotypic characterisation of T(CMV) cells, CD8^+^ T cells were enriched from PBMCs using the untouched CD8^+^ T Cell Isolation Kit (Miltenyi Biotec) according to the manufacturer’s instructions. Next, the enriched CD8^+^ T cells were stimulated with the CMVpp65 overlapping peptide pool PepTivator CMVpp65 (Miltenyi Biotec) at a concentration of 1 μg/ml of each peptide for 4 hours. Subsequently, IFN-γ-secreting cells were detected using IFN-γ Secretion Assay/Detection Kit (PE) (Miltenyi Biotec) according to the manufacturer’s instructions and subsequently phenotypically characterised by flow cytometry.

### Isolation of CD8^+^ T cell subsets for WGBS, pyrosequencing, qRT-PCR, and *in vitro* cultivation experiments

For the isolation of CD8^+^ T cell subsets for WGBS, PBMCs were first enriched for CD8^+^ T cells and subsequently stimulated with CMVpp65 overlapping peptide pool as described above. After the detection of IFN-γ-secreting cells, cells were stained and T(CMV) cells were isolated by flow cytometry-based cell sorting as CD3^+^CD4^−^CD8^+^IFN-γ^+^ cells, while memory CD8^+^ T cells (T_mem_; CD3^+^CD4^−^CD8^+^CCR7^−^CD28^high^CD27^+^CD45RA^−^) and naive CD8^+^ T cells (T_N_; CD3^+^CD4^−^CD8^+^CCR7^+^CD28^int^KLRG1^−^CX3CR1^−^CD45RA^high^) were sorted as controls.

For pyrosequencing, qRT-PCR and *in vitro* cultivation experiments, human CD8^+^ T cell subsets were isolated from cryopreserved PBMCs. First, CD8^+^ T cells were enriched using human anti-CD8 MicroBeads and the automated magnetic activated cell sorting (autoMACS) system (both Miltenyi Biotec) according to the manufacturer’s instructions. Subsequently, the CD8^+^ T cells were stained and sorted into T_N_ (CD45RA^+^CCR7^+^), T_SCM_ (CD45RA^+^CCR7^+^CD28^+^CD62L^+^CD95^+^), T_CM_ (CD45RA^−^CCR7^+^), T_EM_ (CD45RA^−^CCR7^−^), and T_EMRA_ (CD45RA^+^CCR7^−^) CD8^+^CD3^+^ T cell subsets by flow cytometry.

### Whole-genome bisulfite sequencing (WGBS)

For WGBS, sorted T_N_, T_mem_, and T(CMV) cells from the blood of various donors were used to prepare genomic DNA with the DNeasy Blood & Tissue Kit (Qiagen). Approximately 50 ng genomic double-stranded DNA per donor was converted with sodium bisulfite using the EZ DNA Methylation-Lightning Kit (Zymo Research) and fragmented by sonication (Covaris S220, 10% duty cycle, 175W peak incident power, intensity 5, 200 cycles per burst, 120 seconds). The fragmented DNA served as input for the Accel-NGS Methyl-Seq DNA Library Kit (Swift Biosciences) and resulted in libraries that were sequenced on an Illumina NovaSeq 6000 with depths between 216 and 298 million paired end reads (2 x 100 bp).

Sequencing data were processed using the nf-core/methylseq pipeline (version 2.2.0), using default parameters, genome assembly GRCh38 and—accel = true [91] (software freely accessible at http://doi.org/10.5281/zenodo.1343417). Briefly, the pipeline employs FASTQC (version 0.11.9), trimgalore (version 0.6.7) and bismark (version 0.24.0) [92] for read-level quality control, adapter trimming, bisulfite-aware alignment, and cytosine-level DNA methylation quantification.

Methylation calls were further processed using RnBeads (version 2.17.0) [93] using a minimum per CpG coverage of 2 and removing CpGs that were covered in less than 50% of the samples, that overlapped annotated SNPs, or that were located on sex chromosomes. Genome-wide methylation values were computed by coverage weighted aggregation of CpG-level methylation values across samples in groups for 1 kb and 10 kb tiling windows. Dimensionality reduction using principal component analysis (PCA) was performed on those 50,000 1 kb tiling windows that exhibited the highest variance in aggregate methylation levels across the dataset.

Pairwise DMRs between the groups were identified using Bsmooth/BSseq (version 1.30.0) [94]. Briefly, we applied the Bsmooth() command with default parameters on unfiltered CpG methylation calls. Subsequently, only CpGs with a coverage of at least 5 in two or more samples per group were retained. Based on these filtered CpGs, we computed *t*-statistics and DMRs using BSmooth.tstat (…, estimate.var = "same", local.correct = TRUE) and dmrFinder (…, qcutoff = c(0.01,0.99), maxGap = 200). Gene relations to nearest genes were annotated using GENCODE (version 44). Finally, DMRs containing at least 3 CpGs were selected using an absolute mean methylation level difference of 25%.

Sequencing data and methylation levels reported in this paper were uploaded to GEO under accession number GSE245832. Identified DMRs are listed in **S1 Table**.

### *In vitro* cultivation of CD8^+^ T cell subsets

For *in vitro* cultivation, sorted T_N_ and T_EMRA_ cells were stimulated with plate-bound anti-human CD3 (OKT3; 1 μg/ml; BioLegend) and anti-human CD28 (CD28.2; 0.5 μg/ml; BioLegend) antibodies and cultured in TexMACS medium supplemented with 100 IU/ml human IL-2 at 37°C with 5% CO_2_. Every 5 days during the 30-day culture period, cells were harvested and washed, and an aliquot was taken for pyrosequencing and phenotypic characterisation by flow cytometry. The remaining cells were re-stimulated with plate-bound anti-human CD3 and anti-human CD28 (both 0.5 μg/ml) antibodies and cultured in fresh TexMACS medium supplemented with 100 IU/ml human IL-2.

### Pyrosequencing of the *TBKBP1* DMR

Genomic DNA from *ex vivo* isolated CD8^+^ T cell subsets or *in vitro* cultured cells was extracted using the DNeasy Blood & Tissue Kits (Qiagen) and subsequently bisulfite-converted by using the EZ DNA Methylation-Lightning Kit (Zymo Research) according to the manufacturers’ recommendations. *TBKBP1* DMR was pyrosequenced as described previously [95]. For amplification of the *TBKBP1* DMR and subsequent pyrosequencing, we used the following primers:

‘forward’ 5’-TATTTAAGTTTGGGTGATAGAGTAAGAT-3’

‘reverse’ 5’-Bio-CCCAACCCTCAAAAATATAATATCT-3’

‘sequencing 1’ 5’-GGTGGTGTATGTTTGTAAT-3’

‘sequencing 2’ 5’-GGTTGAGGTGAGTTAAGAT-3’

### Quantitative RT-PCR of *TBKBP1*

Total RNA was purified from isolated CD8^+^ T cell subsets using the RNeasy Mini Kit (Qiagen), spectrometrically quantified (DeNovix), and transcribed into cDNA using Transcriptor First Strand cDNA Synthesis Kit (Roche). TBKBP1-A primers (‘forward’ 5’-TTGCCCTCATCACTGCTTAC-3’; ‘reverse’ 5’-GGGTACTTGATCTCGTAGACTTTG-3’), TBKBP1-B primers (‘forward’ 5’-CAGGATCTGGCCTCCAAC-3’; ‘reverse’ 5’-CCCTGTAGGGAACTCAACTC-3’) or codon-optimized TBKBP1 primers (‘forward’ 5’-TCGCTCTCATCACTGCCTAC-3’; ‘reverse’ 5’-GGGTACTTGATTTCATAGACTTTA-3’) and SYBR green master mix (Roche) as well as cDNA were used in a qRT-PCR on a LightCycler 480 II (Roche) or QuantStudio 3 (Thermo Fisher Scientific). The data were analysed with LightCycler 96 SW 1.1 (Roche) or Design & Analysis Software v2.7.0 (Thermo Fisher Scientific). All procedures were performed according to the manufacturers’ recommendations.

### Retroviral transduction

For retroviral overexpression of TBKBP1, an expression cassette consisting of codon-optimized *TBKBP1* cDNA, porcine teschovirus-1 2A element (P2A), and mCherry cDNA was inserted into the pMP71 plasmid by gene synthesis (TWIST Bioscience). pMP71 containing only mCherry cDNA served as empty vector (EV) control. For the ARMATA, cells were not only retrovirally transduced with the TBKBP1 overexpression construct (or the EV control), but also with a pMP71-based plasmid coding for mTCR 5–2, a previously reported CMV-specific, HLA-A*02:01-restricted high-avidity TCR recognising the CMVpp65-derived peptide NLVPMVATV and containing a murine constant region (mTCR) for the isolation of successfully transduced cells [70].

Virus transduction of human PBMCs was performed as previously described [96]. In brief, the virus-packaging RD114 cell line was seeded in a 6-well plate at a density of 1.5 x 10^6^ cells/well 1 day prior to transfection. The next day, pMP71 vectors were transfected using calcium phosphate precipitation. Thereto, complete DMEM medium (Gibco) supplemented with 10% FBS (Gibco), 1% sodium pyruvate (Biochrom), and 1% HEPES (Sigma-Aldrich) was replaced 1 hour before transfection with complete medium containing 25 μM chloroquine diphosphate salt (Sigma-Aldrich). The plasmid-containing buffer was prepared by adding 18 μg of vector DNA and 15 μl of 2.5 M CaCl_2_ (Applichem) to a final volume of 150 μl with water, which was then mixed with transfection buffer containing 280 mM sodium chloride, 42 mM HEPES, 3.5 mM disodium hydrogen phosphate, and 10 mM potassium chloride at 1:1 ratio by vortexing and subsequently incubated for 30 minutes at room temperature. The mixture was slowly added to RD114 cells (150 μl/well) in a dropwise manner and incubated for 15 hours at 37°C with 5% CO_2_, followed by medium replacement with 3 ml complete DMEM medium. The supernatant containing retroviral particles was harvested 3 days later. For transduction, PBMCs were stimulated for 48 hours with plate-bound anti-human CD3 and anti-human CD28 (both 1 μg/ml). Additionally, retronectin-treated plates were prepared by coating with 12.5 μg/ml retronectin (TaKaRa) in PBS (Gibco) at 4°C overnight in a non-treated 24-well plate. Unbound retronectin was aspirated and plates were blocked with 2% BSA (Sigma-Aldrich) in PBS for 20 minutes at 37°C followed by washing twice with PBS. Supernatant containing retroviral particles was added to retronectin-coated plates followed by centrifugation at 2,000xg for 2 hours at 32°C. Following centrifugation, virus supernatant was aspirated and preactivated PBMCs (0.5–0.8 x 10^6^/well) in TexMACS medium supplemented with 100 IU/ml IL-2 and 6 ng/ml polybrene (Sigma-Aldrich) were added to each well. After spinoculating the cells onto virus-coated plates (1,000xg for 10 minutes at 32°C), the cells were incubated at 37°C with 5% CO_2_ for 24 hours. After 24 hours, transduced human PBMCs were washed and resuspended in TexMACS medium supplemented with IL-2. Flow cytometry analysis was performed 5 days after spinoculation to determine transfection efficiency. Successfully transduced CD8^+^mCherry^+^ T cells (TBKBP1 overexpression or EV control) or CD8^+^mTCR^+^mCherry^+^ T cells (simultaneous overexpression of mTCR 5–2 and TBKBP1 or EV control) were sorted using flow cytometry and used for subsequent experiments.

### Cell stimulation and immunoblotting

For sodium dodecyl sulphate polyacrylamide gel electrophoresis (SDS-PAGE) and subsequent immunoblotting experiments, TBKBP1-overexpressing CD8^+^ T cells and control cells transduced with EV as well as *ex vivo* isolated CD8^+^ T_N_, T_CM_, T_EM_ and T_EMRA_ cells were generated and sorted as described above. Next, cells were serum-starved overnight and subsequently incubated with soluble 2 μg/ml anti-CD3 and anti-CD28 mAbs for 40 minutes on ice. Antibody-coated cells were then cross-linked with 4 μg/ml AffiniPure goat-anti-mouse IgG+IgM (H+L; Jackson ImmunoResearch) and incubated for 15 minutes at 37°C. Unstimulated controls were kept on ice. To stop the stimulation, excess ice-cold PBS was added and the cells were centrifuged at 4°C. Pellets were snap-frozen and stored at -80°C.

For preparing cell lysates from TBKBP1-overexpressing CD8^+^ T cells and control cells transduced with EV for SDS-PAGE and immunoblotting, cell pellets were resuspended in 40 μl of 2x SDS loading buffer (0.125 M Tris-HCl, pH 6.8, 4% SDS, 20% glycerol, 0.02% bromphenol blue, 10% β-mercaptoethanol), heated at 95°C for 5 minutes, and cell debris was pelleted with 14,000xg at room temperature (RT) for 5 minutes. Per lane, 20 μl lysate was analysed on 10% polyacrylamide gels containing 0.1% SDS in Tris-glycine running buffer with 0.1% SDS at room temperature.

For preparing cell lysates from *ex vivo* isolated CD8^+^ T cell subsets for SDS-PAGE and immunoblotting, approximately 1 x 10^6^ cells were resuspended in 20 μl lysis buffer (20 mM Tris-base, 100 mM NaCl, 1 mM EDTA, 1% Triton X-100) supplemented with protease and phosphatase inhibitors (both from Roche). Cells were lysed for 1h at 4°C and cell debris was precipitated at 14,000xg at 4°C for 5 minutes. Protein concentration was assessed colorimetrically via the bicinchoninic acid (BCA) protein assay (Takara), adjusted protein amounts were mixed with 4x SDS loading buffer (0.25 M Tris-HCl, pH 6.8, 8% SDS, 40% glycerol, 0.04% bromphenol blue, 10% β-mercaptoethanol), lysates were heated at 95°C for 10 minutes, and loaded on 7.5% polyacrylamide gels containing 0.1% SDS in Tris-glycine running buffer with 0.1% SDS at room temperature.

Proteins were blotted on polyvinylidene difluoride membranes (Amersham Hybond P 0.2 μm) in a wet blot system in Tris-glycine transfer buffer containing 0.05% SDS and 20% methanol for 1 hour at 350 mA at 4°C. Membranes were subsequently blocked in 5% BSA in TBS with 0.1% Tween 20 for 1 hour at room temperature and incubated with anti-phospho-TBK1/NAK (Ser 172; Cell Signaling Technology), anti-TBK1/NAK (D1B4; Cell Signaling Technology) anti-SINTBAD (TBKBP1; D1A5; Cell Signaling Technology), anti-GAPDH (14C10; Cell Signaling Technology) or anti-α-Tubulin (Sigma-Aldrich) primary antibodies at 4°C overnight. Membranes were washed and incubation with HRP-coupled anti-rabbit or anti-mouse secondary antibodies (both from Dianova) was carried out for 1 hour at room temperature. Membranes were washed and developed with the Pierce ECL or SuperSignal substrate (Thermo Fisher Scientific) and a ChemoStar ECL Imager (INTAS). Quantitative analysis was performed with ImageJ software (version 1.52g, National Institute of Health). When necessary, membranes were stripped with Restore PLUS Western Blot Stripping Buffer (ThermoFisher Scientific) for 15 minutes at room temperature, before being washed, blocked, incubated with primary and secondary antibodies, imaged, and analysed as described above.

### PKCθ inhibition

PKCθ inhibition experiments were carried out with EV-transduced or TBKBP1-overexpressing CD8^+^ T cells generated as described above. To assess the impact of PKCθ inhibition on TBK1 phosphorylation, sorted CD8^+^mCherry^+^ T cells were serum-starved overnight and subsequently preincubated for 1 hour with the PKCθ inhibitor (10 μM HY-112681, Hoelzel Biotech). Next, cells were restimulated for 15 minutes and further processed for SDS-PAGE and immunoblotting as described above. To assess the impact of PKCθ inhibition on the functional properties of CD8^+^ T cells, sorted CD8^+^mCherry^+^ T cells were preincubated for 1 hour with the PKCθ inhibitor as described above. DMSO-treated cells were used as control. Next, cells were stimulated for 2 hours using T cell TransACT (Miltenyi Biotech) as recommended by the manufacturer. Afterwards, Brefeldin A (10 μg/ml, Sigma-Aldrich) was added and cells were cultivated for additional 2 hours. At the end of the culture, cells were harvested and stained for flow cytometry as described above.

### Assay for rapid measurement of antiviral T cell activity

For ARMATA [68], CD8^+^ T cells simultaneously overexpressing mTCR 5–2 and TBKBP1 (or the EV control) were generated and sorted as described above. One day prior to infection, MRC-5 cells were seeded in 96-well plates at a density of 20,000 cells/well and cultured in Eagle’s minimum essential medium with 10% FCS, 1 mM sodium pyruvate, and 1 mM glutamine. The following day, the MRC-5 cells were infected with a reporter CMV (TB40/BAC4 HCMV^3F^) that was generated on the background of the clinical isolate TB40-E [69]. Briefly, after aspiration of the medium, the reporter CMV was added to the MRC-5 cells at a MOI of 0.1 and centrifuged at 700xg for 10 minutes at room temperature to enhance the infection and reduce infection heterogeneity. After centrifugation, the medium was immediately aspirated and the cells were washed with PBS. After washing, sorted mTCR^+^mCherry^+^ CD8^+^ T cells were added to the infected MRC-5 cells in a final volume of 200 μl medium at effector:target (E:T) ratios of 0.5:1 and 1:1. Coculture medium was composed of CTS OpTmizer T-cell expansion basal medium (ThermoFischer Scientific) supplemented with CTS Immune Cell SR (ThermoFischer Scientific) and 300 IU/ml IL-2. To analyse the virus reduction capacity of the T cells, the mNeonGreen signals from infected MRC-5 cells were monitored for 1 week using the Incucyte Live-Cell analysis system (Sartorius). Additionally, the confluence of cells was determined by the phase detector and images were analysed by the Incucyte Analysis Software. Data were exported to Prism 9.4.0 for graphical representation.

### Cytokine multiplex assay

For measuring cytokines, 50 μl culture supernatants were harvested after 36 hours of the co-culture from the ARMATA. Supernatants were centrifuged at 500xg for 15 minutes at 4°C and kept at -80°C. For cytokine measurement, 50 μl thawed supernatant was diluted 1:1 with supplied assay buffer. Cytokines were measured using the MILLIPLEX Human CD8^+^ T Cell Magnetic Bead Panel Premixed 17 Plex-Immunology Multiplex Assay (Millipore) according to the manufacturer’s recommendations and analysis was done using the Bio-Plex Manager 6.1 software based on the standard curve.

### Statistical analysis

Prism 9.4.0 software was utilized for statistical analysis. The normal distribution of data points was checked using the Shapiro-Wilk normality test. A paired two-tailed parametric student’s *t* test was conducted on samples that passed the normality test, and a non-parametric Wilcoxon matched-pairs signed rank test was performed on samples that failed in normality test. All data are presented as mean or mean ± SD, and *p*-values ≤ 0.05 deemed significant (* p ≤ 0.05; ** p ≤ 0.01; *** p ≤ 0.001; **** p ≤ 0.0001; ns; not significant).

## Supporting information

S1 Fig Phenotypic characterisation of T(CMV) cells. PBMCs from healthy CMV-seropositive donors were pre-enriched for CD8^+^ T cells and subsequently stimulated with the CMVpp65 overlapping peptide pool. Next, IFN-γ-secreting cells were detected using the IFN-γ Secretion Assay/Detection Kit. The phenotype of IFN-γ-secreting T(CMV) cells was determined using flow cytometry. **(A)** Representative flow cytometry plots show the identification of T(CMV) cells (left) and the phenotypic characterisation via CD45RA and CD62L expression (right). Numbers indicate frequencies in gates or quadrants. **(B)** The bar plot shows the frequencies of T_N_ (CD45RA^+^CD62L^+^), T_CM_ (CD45RA^−^CD62L^+^), T_EM_ (CD45RA^−^CD62L^−^) and T_EMRA_ cells (CD45RA^+^CD62L^−^) within T(CMV) cells from 4 donors. Black dots indicate frequencies from individual donors and grey bar mean values with SD.
(PDF)

S2 Fig Sorting of CD8^+^ T cell subsets for WGBS. PBMCs from healthy CMV-seropositive donors were pre-enriched for CD8^+^ T cells and stimulated with CMVpp65 overlapping peptide pool to detect IFN-γ-secreting T(CMV) cells using IFN-γ Secretion Assay/Detection Kit. Representative flow cytometric plots show the gating strategy for sorting of T_N_ (CD3^+^CD4^−^CD8^+^CCR7^+^CD28^int^KLRG1^−^CX_3_CR1^−^CD45RA^high^) (orange gate), T_mem_ (CD3^+^CD4^−^CD8^+^CCR7^−^CD28^high^CD27^+^CD45RA^−^) (light blue gate), and T(CMV) cells (CD3^+^CD4^−^CD8^+^IFN-γ^+^) (red gate) from 5 donors.
(PDF)

S3 Fig Correlation between methylation level of *TBKBP1* DMR and *TBKBP1* expression in CD8^+^ T cells from CMV-seronegative donors. **(A)** Mean methylation level of *TBKBP1* DMR in sorted CD8^+^ T cell subsets T_N_, T_SCM_, T_CM_, T_EM_, and T_EMRA_ cells from five CMV-seronegative donors are shown. **(B)** *TBKBP1* expression in indicated CD8^+^ T cell subsets from five CMV-seronegative donors were analysed by RT-PCR. **(C)** Correlation analysis visualize the relation between methylation status of *TBKBP1* DMR (x-axis) and associated *TBKBP1* gene expression (y-axis) including the linear regression line and the Pearson correlation coefficient (r).
(PDF)

S4 Fig Alternative transcripts generated from the *TBKBP1* gene locus. **(A)** Overview of the exon/intron structure of putative transcripts arising from the *TBKBP1* gene locus on chromosome 17 (source: Ensembl GRCh38.p14). White boxes show untranslated exons, whereas black boxes represent translated exons. Ensembl transcript code, amino acid (aa) length of the protein, primer pairs for RT- PCR and the position of the *TBKBP1* DMR are indicated. **(B)** Ratio of normalized RT-PCR signals from amplificated TBKBP1-A and TBKPB1-B using RNA of T_CM_, T_EM_ and T_EMRA_ cells. For statistical analyses, a paired two-tailed student’s t test was conducted with *, p ≤ 0.05.
(PDF)

S5 Fig Phenotypic characterisation of CD8^+^ T_N_ and T_EMRA_ cells during long-term cultivation. CD8^+^ T_N_ and T_EMRA_ cells were sorted from healthy CMV-seropositive donors and cultured up to 30 days with repetitive restimulations using plate-bound anti-human CD3 and anti-human CD28 antibodies. Every 5 days, cells were harvested, washed and an aliquot was collected to determine the expression of CD8, CCR7, CD45RA, CD62L, CD95, and CD28 by flow cytometry. Representative flow cytometry plots from 5 independent cultures are depicted.
(PDF)

S6 Fig Validation of retroviral TBKBP1 overexpression in CD8^+^ T cells. **(A)** Workflow describing the steps involved in sample processing for immunoblotting experiments. Briefly, after 48 hours of activation, PBMCs obtained from CMV-seronegative donors were transduced with pMP71-based vectors. From both empty vector (EV)- and TBKBP1-transduced samples, CD8^+^mCherry^+^ cells were sorted according to the depicted gating strategy and restimulated with anti-human CD3 and anti-human CD28 antibodies for 15 minutes followed by cross-linking with goat anti-mouse IgG, while keeping unstimulated cells as controls. Both unstimulated and restimulated cells from EV and TBKBP1 samples were subjected to immunoblotting to determine the expression levels of TBKBP1, TBK1, pTBK1, and GAPDH. **(B)** Representative flow cytometry plots show gating strategy (left) for the sorting of CD8^+^mCherry^+^ T cells (top: EV control; bottom: TBKBP1 overexpression) and post-sort purity (right) from one out of four donors. **(C)** Bar plots showing the mRNA expression of *TBKBP1* in sorted CD8^+^mCherry^+^ T cells from both TBKBP1-overexpressing samples and EV-transduced controls relative to *RSP9* mRNA expression.
(PDF)

S7 Fig Effect of PKCθ inhibition in CD8^+^ T cells on phosphorylation of TBK1. TBKBP1-overexpressing CD8^+^ T cells and EV-transduced controls were generated, and successfully transduced CD8^+^mCherry^+^ T cells were sorted by flow cytometry, serum-starved overnight, preincubated with the PKCθ inhibitor or DMSO as control, and subsequently short-term stimulated. Samples from EV-transduced or TBKBP1-overexpressing CD8^+^ T cells were subjected to immunoblotting to determine the expression of TBKBP1, pTBK1, and total TBK1. The analysis of α-Tubulin expression served as loading control. Data from two independent donors are depicted.
(PDF)

S8 Fig Gating strategy for sorting of TBKBP1-overexpressing CD8^+^ T cells and EV-transduced controls. PBMCs isolated from healthy CMV-seronegative donors were stimulated with plate-bound anti-human CD3 and anti-human CD28 antibodies and subsequently co-transduced with mTCR and TBKBP1- or EV-mCherry plasmids. Successfully transduced CD8^+^mTCR^+^mCherry^+^ T cells were sorted using flow cytometry. Representative flow cytometry plots from 3 independent donors show the gating strategy for sorting of CD8^+^mTCR^+^mCherry^+^ T cells and post-sort purity.
(PDF)

S9 Fig Quantification of cytokines from the ARMATA. PBMCs isolated from healthy CMV-seronegative donors were stimulated with plate-bound anti-human CD3 and anti-human CD28 antibodies and subsequently co-transduced with mTCR and TBKBP1- or EV-mCherry plasmids. Successfully transduced CD8^+^mTCR^+^mCherry^+^ T cells were sorted from both TBKBP1-overexpressing samples and EV-transduced controls using flow cytometry and co-cultured with CMV-infected MRC-5 cells followed by the ARMATA. 36 hours after infection, culture supernatants were harvested and cytokine profiles determined from cultures of CMV-infected MRC-5 cells without the addition of CD8^+^ T cells (w/o T cells), in the presence of EV-transduced mTCR 5–2^+^ CD8^+^ T cells (EV) or in the presence of TBKBP1-overexpressing mTCR 5–2^+^ CD8^+^ T cells (TBKBP1) with E:T ratios of 0.5:1 (left) and 1:1 (right) for indicated cytokines. Data for IFN-γ, Granzyme A, Granzyme B and Perforin from the E:T ratio of 1:1 are shown in Fig 7. Data from 3 independent experiments with 2 technical replicates each are shown. Black dots indicate frequencies from individual donors and grey bar mean values with SD. For statistical analyses, a paired two-tailed student’s *t* test (leaving out “w/o T cell” group) was conducted with *, p ≤ 0.05 and **, p ≤ 0.01.
(PDF)

S1 Table Identified DMRs including methylation values from all pair-wise comparisons. (XLSX)

## References

[1] Picarda G, Benedict CA. Cytomegalovirus: shape-shifting the immune system. J Immunol. 2018;200(12):3881–9. doi: 10.4049/jimmunol.1800171 29866770 PMC5992493

[2] Takenaka K, Nishida T, Asano-Mori Y, Oshima K, Ohashi K, Mori T, et al. Cytomegalovirus reactivation after allogeneic hematopoietic stem cell transplantation is associated with a reduced risk of relapse in patients with acute myeloid leukemia who survived to day 100 after transplantation: the Japan Society for Hematopoietic Cell Transplantation Transplantation-related Complication working group. Biol Blood Marrow Transplant. 2015;21(11):2008–16. doi: 10.1016/j.bbmt.2015.07.019 26211985

[3] Teira P, Battiwalla M, Ramanathan M, Barrett AJ, Ahn KW, Chen M, et al. Early cytomegalovirus reactivation remains associated with increased transplant-related mortality in the current era: a CIBMTR analysis. Blood. 2016;127(20):2427–38. doi: 10.1182/blood-2015-11-679639 26884374 PMC4874224

[4] Lisboa LF, Kumar D, Wilson LE, Humar A. Clinical utility of cytomegalovirus cell-mediated immunity in transplant recipients with cytomegalovirus viremia. Transplantation. 2012;93(2):195–200. doi: 10.1097/TP.0b013e31823c1cd4 22179399

[5] Tischer S, Priesner C, Heuft HG, Goudeva L, Mende W, Barthold M, et al. Rapid generation of clinical-grade antiviral T cells: selection of suitable T-cell donors and GMP-compliant manufacturing of antiviral T cells. J Transl Med. 2014;12:336. doi: 10.1186/s12967-014-0336-5 25510656 PMC4335407

[6] Link CS, Eugster A, Heidenreich F, Rucker-Braun E, Schmiedgen M, Oelschlagel U, et al. Abundant cytomegalovirus (CMV) reactive clonotypes in the CD8^+^ T cell receptor alpha repertoire following allogeneic transplantation. Clin Exp Immunol. 2016;184(3):389–402. doi: 10.1111/cei.12770 26800118 PMC4872374

[7] Lugthart G, van Ostaijen-Ten Dam MM, Jol-van der Zijde CM, van Holten TC, Kester MG, Heemskerk MH, et al. Early cytomegalovirus reactivation leaves a specific and dynamic imprint on the reconstituting T cell compartment long-term after hematopoietic stem cell transplantation. Biol Blood Marrow Transplant. 2014;20(5):655–61. doi: 10.1016/j.bbmt.2014.01.018 24462981

[8] Suessmuth Y, Mukherjee R, Watkins B, Koura DT, Finstermeier K, Desmarais C, et al. CMV reactivation drives posttransplant T-cell reconstitution and results in defects in the underlying TCRbeta repertoire. Blood. 2015;125(25):3835–50. 25852054 10.1182/blood-2015-03-631853 PMC4473113

[9] Einsele H, Roosnek E, Rufer N, Sinzger C, Riegler S, Loffler J, et al. Infusion of cytomegalovirus (CMV)-specific T cells for the treatment of CMV infection not responding to antiviral chemotherapy. Blood. 2002;99(11):3916–22. doi: 10.1182/blood.v99.11.3916 12010789

[10] Peggs KS, Verfuerth S, Pizzey A, Chow SL, Thomson K, Mackinnon S. Cytomegalovirus-specific T cell immunotherapy promotes restoration of durable functional antiviral immunity following allogeneic stem cell transplantation. Clin Infect Dis. 2009;49(12):1851–60. doi: 10.1086/648422 19911966

[11] Walter EA, Greenberg PD, Gilbert MJ, Finch RJ, Watanabe KS, Thomas ED, et al. Reconstitution of cellular immunity against cytomegalovirus in recipients of allogeneic bone marrow by transfer of T-cell clones from the donor. N Engl J Med. 1995;333(16):1038–44. doi: 10.1056/NEJM199510193331603 7675046

[12] Neuenhahn M, Albrecht J, Odendahl M, Schlott F, Dossinger G, Schiemann M, et al. Transfer of minimally manipulated CMV-specific T cells from stem cell or third-party donors to treat CMV infection after allo-HSCT. Leukemia. 2017;31(10):2161–71. doi: 10.1038/leu.2017.16 28090089

[13] Uhlin M, Gertow J, Uzunel M, Okas M, Berglund S, Watz E, et al. Rapid salvage treatment with virus-specific T cells for therapy-resistant disease. Clin Infect Dis. 2012;55(8):1064–73. doi: 10.1093/cid/cis625 22806594

[14] Hertoghs KM, Moerland PD, van Stijn A, Remmerswaal EB, Yong SL, van de Berg PJ, et al. Molecular profiling of cytomegalovirus-induced human CD8^+^ T cell differentiation. J Clin Invest. 2010;120(11):4077–90. doi: 10.1172/JCI42758 20921622 PMC2964975

[15] Munks MW, Cho KS, Pinto AK, Sierro S, Klenerman P, Hill AB. Four distinct patterns of memory CD8 T cell responses to chronic murine cytomegalovirus infection. J Immunol. 2006;177(1):450–8. doi: 10.4049/jimmunol.177.1.450 16785542

[16] Sierro S, Rothkopf R, Klenerman P. Evolution of diverse antiviral CD8^+^ T cell populations after murine cytomegalovirus infection. Eur J Immunol. 2005;35(4):1113–23. doi: 10.1002/eji.200425534 15756645

[17] Kalia V, Sarkar S, Ahmed R. CD8 T-cell memory differentiation during acute and chronic viral infections. Adv Exp Med Biol. 2010;684:79–95. doi: 10.1007/978-1-4419-6451-9_7 20795542

[18] Joshi NS, Cui W, Chandele A, Lee HK, Urso DR, Hagman J, et al. Inflammation directs memory precursor and short-lived effector CD8^+^ T cell fates via the graded expression of T-bet transcription factor. Immunity. 2007;27(2):281–95. doi: 10.1016/j.immuni.2007.07.010 17723218 PMC2034442

[19] Intlekofer AM, Takemoto N, Wherry EJ, Longworth SA, Northrup JT, Palanivel VR, et al. Effector and memory CD8^+^ T cell fate coupled by T-bet and eomesodermin. Nat Immunol. 2005;6(12):1236–44. doi: 10.1038/ni1268 16273099

[20] Intlekofer AM, Takemoto N, Kao C, Banerjee A, Schambach F, Northrop JK, et al. Requirement for T-bet in the aberrant differentiation of unhelped memory CD8^+^ T cells. J Exp Med. 2007;204(9):2015–21. doi: 10.1084/jem.20070841 17698591 PMC2118697

[21] Hamann D, Baars PA, Rep MH, Hooibrink B, Kerkhof-Garde SR, Klein MR, et al. Phenotypic and functional separation of memory and effector human CD8^+^ T cells. J Exp Med. 1997;186(9):1407–18. doi: 10.1084/jem.186.9.1407 9348298 PMC2199103

[22] Hamann D, Roos MT, van Lier RA. Faces and phases of human CD8 T-cell development. Immunol Today. 1999;20(4):177–80. doi: 10.1016/s0167-5699(99)01444-9 10203715

[23] Henning AN, Roychoudhuri R, Restifo NP. Epigenetic control of CD8^+^ T cell differentiation. Nat Rev Immunol. 2018;18(5):340–56. doi: 10.1038/nri.2017.146 29379213 PMC6327307

[24] Lan X, Zebley CC, Youngblood B. Cellular and molecular waypoints along the path of T cell exhaustion. Sci Immunol. 2023;8(87):eadg3868. doi: 10.1126/sciimmunol.adg3868 37656775 PMC10618911

[25] Frias AB, Boi SK, Lan X, Youngblood B. Epigenetic regulation of T cell adaptive immunity. Immunol Rev. 2021;300(1):9–21. doi: 10.1111/imr.12943 33644866 PMC8005453

[26] Xu T, Pereira RM, Martinez GJ. An updated model for the epigenetic regulation of effector and memory CD8^+^ T cell differentiation. J Immunol. 2021;207(6):1497–505. doi: 10.4049/jimmunol.2100633 34493604

[27] Araki Y, Wang Z, Zang C, Wood WH 3rd, Schones D, Cui K, et al. Genome-wide analysis of histone methylation reveals chromatin state-based regulation of gene transcription and function of memory CD8^+^ T cells. Immunity. 2009;30(6):912–25. doi: 10.1016/j.immuni.2009.05.006 19523850 PMC2709841

[28] Crompton JG, Narayanan M, Cuddapah S, Roychoudhuri R, Ji Y, Yang W, et al. Lineage relationship of CD8^+^ T cell subsets is revealed by progressive changes in the epigenetic landscape. Cell Mol Immunol. 2016;13(4):502–13. doi: 10.1038/cmi.2015.32 25914936 PMC4947817

[29] Russ BE, Olshanksy M, Smallwood HS, Li J, Denton AE, Prier JE, et al. Distinct epigenetic signatures delineate transcriptional programs during virus-specific CD8^+^ T cell differentiation. Immunity. 2014;41(5):853–65. doi: 10.1016/j.immuni.2014.11.001 25517617 PMC4479393

[30] Ball MP, Li JB, Gao Y, Lee JH, LeProust EM, Park IH, et al. Targeted and genome-scale strategies reveal gene-body methylation signatures in human cells. Nat Biotechnol. 2009;27(4):361–8. doi: 10.1038/nbt.1533 19329998 PMC3566772

[31] Jones PA. Functions of DNA methylation: islands, start sites, gene bodies and beyond. Nat Rev Genet. 2012;13(7):484–92. doi: 10.1038/nrg3230 22641018

[32] Scharer CD, Barwick BG, Youngblood BA, Ahmed R, Boss JM. Global DNA methylation remodeling accompanies CD8 T cell effector function. J Immunol. 2013;191(6):3419–29. doi: 10.4049/jimmunol.1301395 23956425 PMC3800465

[33] Rodriguez RM, Suarez-Alvarez B, Lavin JL, Mosen-Ansorena D, Baragano Raneros A, Marquez-Kisinousky L, et al. Epigenetic networks regulate the transcriptional program in memory and terminally differentiated CD8^+^ T cells. J Immunol. 2017;198(2):937–49. doi: 10.4049/jimmunol.1601102 27974453

[34] Abdelsamed HA, Moustaki A, Fan Y, Dogra P, Ghoneim HE, Zebley CC, et al. Human memory CD8 T cell effector potential is epigenetically preserved during in vivo homeostasis. J Exp Med. 2017;214(6):1593–606. doi: 10.1084/jem.20161760 28490440 PMC5461005

[35] Ji H, Ehrlich LI, Seita J, Murakami P, Doi A, Lindau P, et al. Comprehensive methylome map of lineage commitment from haematopoietic progenitors. Nature. 2010;467(7313):338–42. doi: 10.1038/nature09367 20720541 PMC2956609

[36] Meissner A, Mikkelsen TS, Gu H, Wernig M, Hanna J, Sivachenko A, et al. Genome-scale DNA methylation maps of pluripotent and differentiated cells. Nature. 2008;454(7205):766–70. doi: 10.1038/nature07107 18600261 PMC2896277

[37] Stadler MB, Murr R, Burger L, Ivanek R, Lienert F, Scholer A, et al. DNA-binding factors shape the mouse methylome at distal regulatory regions. Nature. 2011;480(7378):490–5. doi: 10.1038/nature10716 22170606

[38] Scott-Browne JP, Lopez-Moyado IF, Trifari S, Wong V, Chavez L, Rao A, et al. Dynamic changes in chromatin accessibility occur in CD8^+^ T cells responding to viral infection. Immunity. 2016;45(6):1327–40. doi: 10.1016/j.immuni.2016.10.028 27939672 PMC5214519

[39] Yu B, Zhang K, Milner JJ, Toma C, Chen R, Scott-Browne JP, et al. Epigenetic landscapes reveal transcription factors that regulate CD8^+^ T cell differentiation. Nat Immunol. 2017;18(5):573–82. doi: 10.1038/ni.3706 28288100 PMC5395420

[40] Akondy RS, Fitch M, Edupuganti S, Yang S, Kissick HT, Li KW, et al. Origin and differentiation of human memory CD8 T cells after vaccination. Nature. 2017;552(7685):362–7. doi: 10.1038/nature24633 29236685 PMC6037316

[41] Zebley CC, Abdelsamed HA, Ghoneim HE, Alli S, Brown C, Haydar D, et al. Proinflammatory cytokines promote TET2-mediated DNA demethylation during CD8 T cell effector differentiation. Cell Rep. 2021;37(2):109796. 34644568 10.1016/j.celrep.2021.109796 PMC8593824

[42] Boppana SB, Britt WJ. Recognition of human cytomegalovirus gene products by HCMV-specific cytotoxic T cells. Virology. 1996;222(1):293–6. doi: 10.1006/viro.1996.0424 8806513

[43] Gordon CL, Miron M, Thome JJ, Matsuoka N, Weiner J, Rak MA, et al. Tissue reservoirs of antiviral T cell immunity in persistent human CMV infection. J Exp Med. 2017;214(3):651–67. doi: 10.1084/jem.20160758 28130404 PMC5339671

[44] Schmidt F, Fields HF, Purwanti Y, Milojkovic A, Salim S, Wu KX, et al. In-depth analysis of human virus-specific CD8^+^ T cells delineates unique phenotypic signatures for T cell specificity prediction. Cell Rep. 2023;42(10):113250. 37837618 10.1016/j.celrep.2023.113250

[45] Durek P, Nordstrom K, Gasparoni G, Salhab A, Kressler C, de Almeida M, et al. Epigenomic profiling of human CD4^+^ T cells supports a linear differentiation model and highlights molecular regulators of memory development. Immunity. 2016;45(5):1148–61. doi: 10.1016/j.immuni.2016.10.022 27851915

[46] Ghoneim HE, Fan Y, Moustaki A, Abdelsamed HA, Dash P, Dogra P, et al. De novo epigenetic programs inhibit PD-1 blockade-mediated T cell rejuvenation. Cell. 2017;170(1):142–57 e19. doi: 10.1016/j.cell.2017.06.007 28648661 PMC5568784

[47] van Stijn A, Rowshani AT, Yong SL, Baas F, Roosnek E, ten Berge IJ, et al. Human cytomegalovirus infection induces a rapid and sustained change in the expression of NK cell receptors on CD8^+^ T cells. J Immunol. 2008;180(7):4550–60. doi: 10.4049/jimmunol.180.7.4550 18354177

[48] Mayer KD, Mohrs K, Reiley W, Wittmer S, Kohlmeier JE, Pearl JE, et al. Cutting edge: T-bet and IL-27R are critical for in vivo IFN-gamma production by CD8 T cells during infection. J Immunol. 2008;180(2):693–7. doi: 10.4049/jimmunol.180.2.693 18178806

[49] Giles JR, Ngiow SF, Manne S, Baxter AE, Khan O, Wang P, et al. Shared and distinct biological circuits in effector, memory and exhausted CD8^+^ T cells revealed by temporal single-cell transcriptomics and epigenetics. Nat Immunol. 2022;23(11):1600–13. doi: 10.1038/s41590-022-01338-4 36271148 PMC10408358

[50] Evrard M, Wynne-Jones E, Peng C, Kato Y, Christo SN, Fonseca R, et al. Sphingosine 1-phosphate receptor 5 (S1PR5) regulates the peripheral retention of tissue-resident lymphocytes. J Exp Med. 2022;219(1):e20210116. doi: 10.1084/jem.20210116 34677611 PMC8546662

[51] Ryzhakov G, Randow F. SINTBAD, a novel component of innate antiviral immunity, shares a TBK1-binding domain with NAP1 and TANK. EMBO J. 2007;26(13):3180–90. doi: 10.1038/sj.emboj.7601743 17568778 PMC1914091

[52] Zhu L, Li Y, Xie X, Zhou X, Gu M, Jie Z, et al. TBKBP1 and TBK1 form a growth factor signalling axis mediating immunosuppression and tumourigenesis. Nat Cell Biol. 2019;21(12):1604–14. doi: 10.1038/s41556-019-0429-8 31792381 PMC6901116

[53] Runde AP, Mack R, S JP, Zhang J. The role of TBK1 in cancer pathogenesis and anticancer immunity. J Exp Clin Cancer Res. 2022;41(1):135. doi: 10.1186/s13046-022-02352-y 35395857 PMC8994244

[54] Honda K, Takaoka A, Taniguchi T. Type I interferon [corrected] gene induction by the interferon regulatory factor family of transcription factors. Immunity. 2006;25(3):349–60. doi: 10.1016/j.immuni.2006.08.009 16979567

[55] Takeuchi O, Akira S. Pattern recognition receptors and inflammation. Cell. 2010;140(6):805–20. doi: 10.1016/j.cell.2010.01.022 20303872

[56] Fitzgerald KA, McWhirter SM, Faia KL, Rowe DC, Latz E, Golenbock DT, et al. IKKepsilon and TBK1 are essential components of the IRF3 signaling pathway. Nat Immunol. 2003;4(5):491–6. doi: 10.1038/ni921 12692549

[57] Hemmi H, Takeuchi O, Sato S, Yamamoto M, Kaisho T, Sanjo H, et al. The roles of two IkappaB kinase-related kinases in lipopolysaccharide and double stranded RNA signaling and viral infection. J Exp Med. 2004;199(12):1641–50. doi: 10.1084/jem.20040520 15210742 PMC2212809

[58] Hiscott J. Triggering the innate antiviral response through IRF-3 activation. J Biol Chem. 2007;282(21):15325–9. doi: 10.1074/jbc.R700002200 17395583

[59] Sharma S, tenOever BR, Grandvaux N, Zhou GP, Lin R, Hiscott J. Triggering the interferon antiviral response through an IKK-related pathway. Science. 2003;300(5622):1148–51. doi: 10.1126/science.1081315 12702806

[60] Anastasiadi D, Esteve-Codina A, Piferrer F. Consistent inverse correlation between DNA methylation of the first intron and gene expression across tissues and species. Epigenetics Chromatin. 2018;11(1):37. doi: 10.1186/s13072-018-0205-1 29958539 PMC6025724

[61] de Mendoza A, Nguyen TV, Ford E, Poppe D, Buckberry S, Pflueger J, et al. Large-scale manipulation of promoter DNA methylation reveals context-specific transcriptional responses and stability. Genome Biol. 2022;23(1):163. doi: 10.1186/s13059-022-02728-5 35883107 PMC9316731

[62] Li L, Gao Y, Wu Q, Cheng ASL, Yip KY. New guidelines for DNA methylome studies regarding 5-hydroxymethylcytosine for understanding transcriptional regulation. Genome Res. 2019;29(4):543–53. doi: 10.1101/gr.240036.118 30782641 PMC6442395

[63] Lev Maor G, Yearim A, Ast G. The alternative role of DNA methylation in splicing regulation. Trends Genet. 2015;31(5):274–80. doi: 10.1016/j.tig.2015.03.002 25837375

[64] Zhu L, Xie X, Zhang L, Wang H, Jie Z, Zhou X, et al. TBK-binding protein 1 regulates IL-15-induced autophagy and NKT cell survival. Nat Commun. 2018;9(1):2812. doi: 10.1038/s41467-018-05097-5 30022064 PMC6052109

[65] Gattinoni L, Lugli E, Ji Y, Pos Z, Paulos CM, Quigley MF, et al. A human memory T cell subset with stem cell-like properties. Nat Med. 2011;17(10):1290–7. doi: 10.1038/nm.2446 21926977 PMC3192229

[66] Wherry EJ, Teichgraber V, Becker TC, Masopust D, Kaech SM, Antia R, et al. Lineage relationship and protective immunity of memory CD8 T cell subsets. Nat Immunol. 2003;4(3):225–34. doi: 10.1038/ni889 12563257

[67] Yu J, Zhou X, Chang M, Nakaya M, Chang JH, Xiao Y, et al. Regulation of T-cell activation and migration by the kinase TBK1 during neuroinflammation. Nat Commun. 2015;6:6074. doi: 10.1038/ncomms7074 25606824 PMC4302769

[68] Khan F, Muller TR, Kasmapour B, Ynga-Durand MA, Eiz-Vesper B, von Einem J, et al. Dynamic monitoring of viral gene expression reveals rapid antiviral effects of CD8 T cells recognizing the HCMV-pp65 antigen. Front Immunol. 2024;15:1439184. doi: 10.3389/fimmu.2024.1439184 39104541 PMC11299495

[69] Rand U, Kubsch T, Kasmapour B, Cicin-Sain L. A novel triple-fluorescent HCMV strain reveals gene expression dynamics and anti-herpesviral drug mechanisms. Front Cell Infect Microbiol. 2020;10:536150. doi: 10.3389/fcimb.2020.536150 33489928 PMC7820782

[70] Schober K, Voit F, Grassmann S, Muller TR, Eggert J, Jarosch S, et al. Reverse TCR repertoire evolution toward dominant low-affinity clones during chronic CMV infection. Nat Immunol. 2020;21(4):434–41. doi: 10.1038/s41590-020-0628-2 32205883

[71] Tian Y, Babor M, Lane J, Seumois G, Liang S, Goonawardhana NDS, et al. Dengue-specific CD8^+^ T cell subsets display specialized transcriptomic and TCR profiles. J Clin Invest. 2019;129(4):1727–41. doi: 10.1172/JCI123726 30882366 PMC6436856

[72] Wilson CB, Rowell E, Sekimata M. Epigenetic control of T-helper-cell differentiation. Nat Rev Immunol. 2009;9(2):91–105. doi: 10.1038/nri2487 19151746

[73] Youngblood B, Oestreich KJ, Ha SJ, Duraiswamy J, Akondy RS, West EE, et al. Chronic virus infection enforces demethylation of the locus that encodes PD-1 in antigen-specific CD8^+^ T cells. Immunity. 2011;35(3):400–12. doi: 10.1016/j.immuni.2011.06.015 21943489 PMC3183460

[74] Quezada LK, Jin W, Liu YC, Kim ES, He Z, Indralingam CS, et al. Early transcriptional and epigenetic divergence of CD8^+^ T cells responding to acute versus chronic infection. PLoS Biol. 2023;21(1):e3001983. doi: 10.1371/journal.pbio.3001983 36716323 PMC9886247

[75] Dominguez CX, Amezquita RA, Guan T, Marshall HD, Joshi NS, Kleinstein SH, et al. The transcription factors ZEB2 and T-bet cooperate to program cytotoxic T cell terminal differentiation in response to LCMV viral infection. J Exp Med. 2015;212(12):2041–56. doi: 10.1084/jem.20150186 26503446 PMC4647261

[76] Omilusik KD, Best JA, Yu B, Goossens S, Weidemann A, Nguyen JV, et al. Transcriptional repressor ZEB2 promotes terminal differentiation of CD8^+^ effector and memory T cell populations during infection. J Exp Med. 2015;212(12):2027–39. doi: 10.1084/jem.20150194 26503445 PMC4647262

[77] van de Berg PJ, Yong SL, Remmerswaal EB, van Lier RA, ten Berge IJ. Cytomegalovirus-induced effector T cells cause endothelial cell damage. Clin Vaccine Immunol. 2012;19(5):772–9. doi: 10.1128/CVI.00011-12 22398244 PMC3346330

[78] Abdel-Hakeem MS, Manne S, Beltra JC, Stelekati E, Chen Z, Nzingha K, et al. Epigenetic scarring of exhausted T cells hinders memory differentiation upon eliminating chronic antigenic stimulation. Nat Immunol. 2021;22(8):1008–19. doi: 10.1038/s41590-021-00975-5 34312545 PMC8323971

[79] Turk L, Filippov I, Arnold C, Zaugg J, Tserel L, Kisand K, et al. Cytotoxic CD8^+^ Temra cells show loss of chromatin accessibility at genes associated with T cell activation. Front Immunol. 2024;15:1285798. doi: 10.3389/fimmu.2024.1285798 38370415 PMC10870784

[80] Mackay LK, Braun A, Macleod BL, Collins N, Tebartz C, Bedoui S, et al. Cutting edge: CD69 interference with sphingosine-1-phosphate receptor function regulates peripheral T cell retention. J Immunol. 2015;194(5):2059–63. doi: 10.4049/jimmunol.1402256 25624457

[81] Mackay LK, Rahimpour A, Ma JZ, Collins N, Stock AT, Hafon ML, et al. The developmental pathway for CD103^+^CD8^+^ tissue-resident memory T cells of skin. Nat Immunol. 2013;14(12):1294–301. doi: 10.1038/ni.2744 24162776

[82] Al Hamrashdi M, Brady G. Regulation of IRF3 activation in human antiviral signaling pathways. Biochem Pharmacol. 2022;200:115026. doi: 10.1016/j.bcp.2022.115026 35367198

[83] Honda K, Yanai H, Negishi H, Asagiri M, Sato M, Mizutani T, et al. IRF-7 is the master regulator of type-I interferon-dependent immune responses. Nature. 2005;434(7034):772–7. doi: 10.1038/nature03464 15800576

[84] Hong Y, Bai M, Qi X, Li C, Liang M, Li D, et al. Suppression of the IFN-alpha and -beta induction through sequestering IRF7 into viral inclusion bodies by nonstructural protein NSs in severe fever with thrombocytopenia syndrome Bunyavirus infection. J Immunol. 2019;202(3):841–56. 30598516 10.4049/jimmunol.1800576

[85] Farlik M, Rapp B, Marie I, Levy DE, Jamieson AM, Decker T. Contribution of a TANK-binding kinase 1-interferon (IFN) regulatory factor 7 pathway to IFN-gamma-induced gene expression. Mol Cell Biol. 2012;32(6):1032–43. 22252317 10.1128/MCB.06021-11 PMC3295005

[86] tenOever BR, Sharma S, Zou W, Sun Q, Grandvaux N, Julkunen I, et al. Activation of TBK1 and IKKvarepsilon kinases by vesicular stomatitis virus infection and the role of viral ribonucleoprotein in the development of interferon antiviral immunity. J Virol. 2004;78(19):10636–49. doi: 10.1128/JVI.78.19.10636-10649.2004 15367631 PMC516426

[87] Moore TC, Vogel AJ, Petro TM, Brown DM. IRF3 deficiency impacts granzyme B expression and maintenance of memory T cell function in response to viral infection. Microbes Infect. 2015;17(6):426–39. doi: 10.1016/j.micinf.2015.03.001 25777301 PMC4479197

[88] Sukdolak C, Tischer S, Dieks D, Figueiredo C, Goudeva L, Heuft HG, et al. CMV-, EBV- and ADV-specific T cell immunity: screening and monitoring of potential third-party donors to improve post-transplantation outcome. Biol Blood Marrow Transplant. 2013;19(10):1480–92. doi: 10.1016/j.bbmt.2013.07.015 23891747

[89] Tischer S, Dieks D, Sukdolak C, Bunse C, Figueiredo C, Immenschuh S, et al. Evaluation of suitable target antigens and immunoassays for high-accuracy immune monitoring of cytomegalovirus and Epstein-Barr virus-specific T cells as targets of interest in immunotherapeutic approaches. J Immunol Methods. 2014;408:101–13. doi: 10.1016/j.jim.2014.05.011 24877879

[90] Cossarizza A, Chang HD, Radbruch A, Abrignani S, Addo R, Akdis M, et al. Guidelines for the use of flow cytometry and cell sorting in immunological studies (third edition). Eur J Immunol. 2021;51(12):2708–3145. doi: 10.1002/eji.202170126 34910301 PMC11115438

[91] Ewels PA, Peltzer A, Fillinger S, Patel H, Alneberg J, Wilm A, et al. The nf-core framework for community-curated bioinformatics pipelines. Nat Biotechnol. 2020;38(3):276–8. doi: 10.1038/s41587-020-0439-x 32055031

[92] Krueger F, Andrews SR. Bismark: a flexible aligner and methylation caller for Bisulfite-Seq applications. Bioinformatics. 2011;27(11):1571–2. doi: 10.1093/bioinformatics/btr167 21493656 PMC3102221

[93] Muller F, Scherer M, Assenov Y, Lutsik P, Walter J, Lengauer T, et al. RnBeads 2.0: comprehensive analysis of DNA methylation data. Genome Biol. 2019;20(1):55. doi: 10.1186/s13059-019-1664-9 30871603 PMC6419383

[94] Hansen KD, Langmead B, Irizarry RA. BSmooth: from whole genome bisulfite sequencing reads to differentially methylated regions. Genome Biol. 2012;13(10):R83. doi: 10.1186/gb-2012-13-10-r83 23034175 PMC3491411

[95] Yang BH, Hagemann S, Mamareli P, Lauer U, Hoffmann U, Beckstette M, et al. Foxp3^+^ T cells expressing RORgammat represent a stable regulatory T-cell effector lineage with enhanced suppressive capacity during intestinal inflammation. Mucosal Immunol. 2016;9(2):444–57. 26307665 10.1038/mi.2015.74

[96] Muller TR, Jarosch S, Hammel M, Leube J, Grassmann S, Bernard B, et al. Targeted T cell receptor gene editing provides predictable T cell product function for immunotherapy. Cell Rep Med. 2021;2(8):100374. 34467251 10.1016/j.xcrm.2021.100374 PMC8385324

