# Supplementary material for: DNA methylation profiling identifies TBKBP1 as potent amplifier of cytotoxic activity in CMV-specific human CD8+ T cells
Source: PLoS Pathog. 2024 Sep 26;20(9):e1012581. doi: 10.1371/journal.ppat.1012581 (PMC11460711; doi:10.1371/journal.ppat.1012581)
Supplement: S1 Fig — PBMCs from healthy CMV-seropositive donors were pre-enriched for CD8+ T cells and subsequently stimulated with the CMVpp65 overlapping peptide pool. Next, IFN-γ-secreting cells were detected using the IFN-γ Secretion Assay/Detection Kit. The phenotype of IFN-γ-secreting T(CMV) cells was determined using flow cytometry. (A) Representative flow cytometry plots show the identification of T(CMV) cells (left) and the phenotypic characterisation via CD45RA and CD62L expression (right). Numbers indicate frequencies in gates or quadrants. (B) The bar plot shows the frequencies of TN (CD45RA+CD62L+), TCM (CD45RA−CD62L+), TEM (CD45RA−CD62L−) and TEMRA cells (CD45RA+CD62L−) within T(CMV) cells from 4 donors. Black dots indicate frequencies from individual donors and grey bar mean values with SD. (PDF) [file ppat.1012581.s001.pdf]

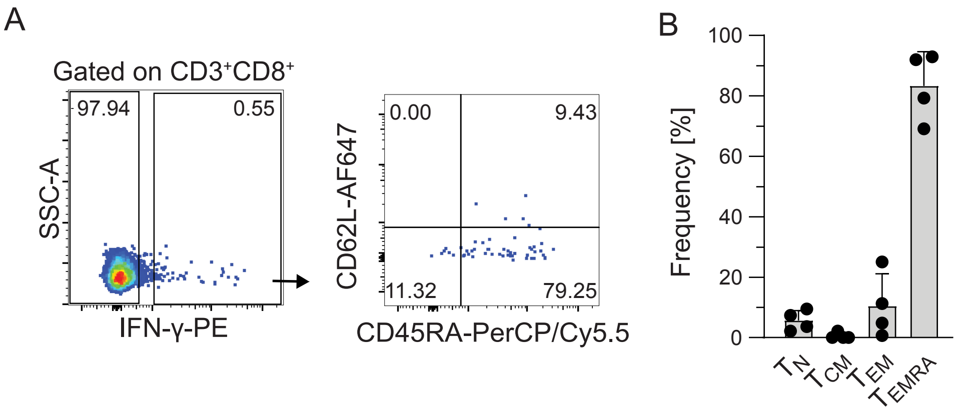


**Supplementary Figure 1: Phenotypic characterisation of T(CMV) cells.** PBMCs from healthy CMV-seropositive donors were pre-enriched for $CD8^+$ T cells and subsequently stimulated with the CMVpp65 overlapping peptide pool. Next, IFN-γ-secreting cells were detected using the IFN-γ Secretion Assay/Detection Kit. The phenotype of IFN-γ-secreting T(CMV) cells was determined using flow cytometry. **(A)** Representative flow cytometry plots show the identification of T(CMV) cells (left) and the phenotypic characterisation via CD45RA and CD62L expression (right). Numbers indicate frequencies in gates or quadrants. **(B)** The bar plot shows the frequencies of $T_N$ ($CD45RA^+CD62L^+$), $T_{CM}$ ($CD45RA^-CD62L^+$), $T_{EM}$ ($CD45RA^-CD62L^-$) and $T_{EMRA}$ cells ($CD45RA^+CD62L^-$) within T(CMV) cells from 4 donors. Black dots indicate frequencies from individual donors and grey bar mean values with SD.
