# Supplementary material for: DNA methylation profiling identifies TBKBP1 as potent amplifier of cytotoxic activity in CMV-specific human CD8+ T cells
Source: PLoS Pathog. 2024 Sep 26;20(9):e1012581. doi: 10.1371/journal.ppat.1012581 (PMC11460711; doi:10.1371/journal.ppat.1012581)
Supplement: S2 Fig — PBMCs from healthy CMV-seropositive donors were pre-enriched for CD8+ T cells and stimulated with CMVpp65 overlapping peptide pool to detect IFN-γ-secreting T(CMV) cells using IFN-γ Secretion Assay/Detection Kit. Representative flow cytometric plots show the gating strategy for sorting of TN (CD3+CD4−CD8+CCR7+CD28intKLRG1−CX3CR1−CD45RAhigh) (orange gate), Tmem (CD3+CD4−CD8+CCR7−CD28highCD27+CD45RA−) (light blue gate), and T(CMV) cells (CD3+CD4−CD8+IFN-γ+) (red gate) from 5 donors. (PDF) [file ppat.1012581.s002.pdf]

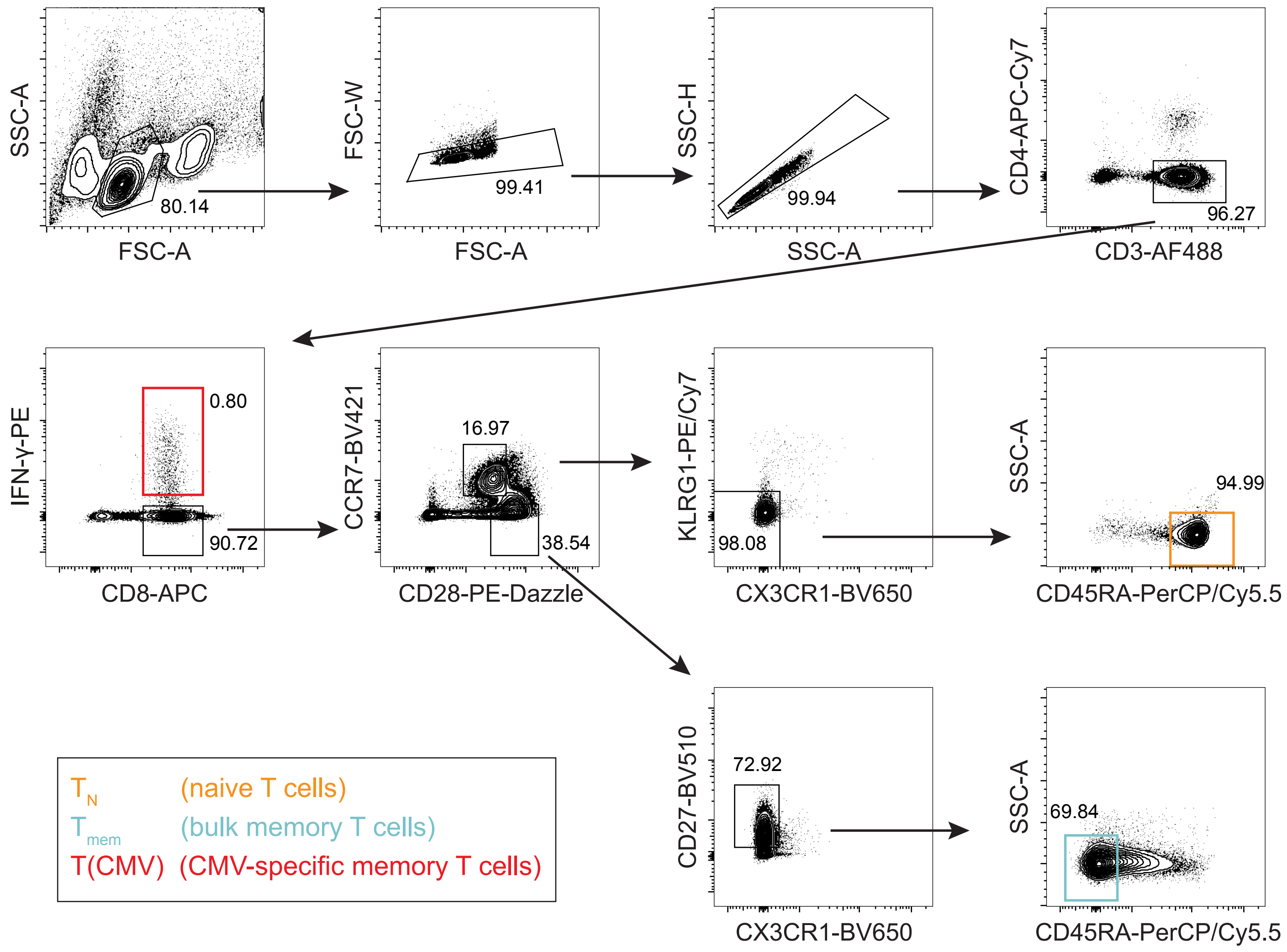

Supplementary Figure 2: Sorting of CD8$^+$ T cell subsets for WGBS. PBMCs from healthy CMV-seropositive donors were pre-enriched for CD8$^+$ T cells and stimulated with CMVpp65 overlapping peptide pool to detect IFN-γ-secreting T(CMV) cells using IFN-γ Secretion Assay/Detection Kit. Representative flow cytometric plots show the gating strategy for sorting of T_N (CD3$^+$CD4$^-$CD8$^+$CCR7$^+$CD28^{int}KLRG1$^-$CX3CR1$^-$CD45RA^{high}) (organge gate), T_{mem} (CD3$^+$CD4$^-$CD8$^+$CCR7$^-$CD28^{high}CD27$^+$CD45RA$^-$) (light blue gate), and T(CMV) cells (CD3$^+$CD4$^-$CD8$^+$IFN-γ$^+$) (red gate) from 5 donors.
