# Supplementary material for: DNA methylation profiling identifies TBKBP1 as potent amplifier of cytotoxic activity in CMV-specific human CD8+ T cells
Source: PLoS Pathog. 2024 Sep 26;20(9):e1012581. doi: 10.1371/journal.ppat.1012581 (PMC11460711; doi:10.1371/journal.ppat.1012581)
Supplement: S3 Fig — (A) Mean methylation level of TBKBP1 DMR in sorted CD8+ T cell subsets TN, TSCM, TCM, TEM, and TEMRA cells from five CMV-seronegative donors are shown. (B) TBKBP1 expression in indicated CD8+ T cell subsets from five CMV-seronegative donors were analysed by RT-PCR. (C) Correlation analysis visualize the relation between methylation status of TBKBP1 DMR (x-axis) and associated TBKBP1 gene expression (y-axis) including the linear regression line and the Pearson correlation coefficient (r). (PDF) [file ppat.1012581.s003.pdf]

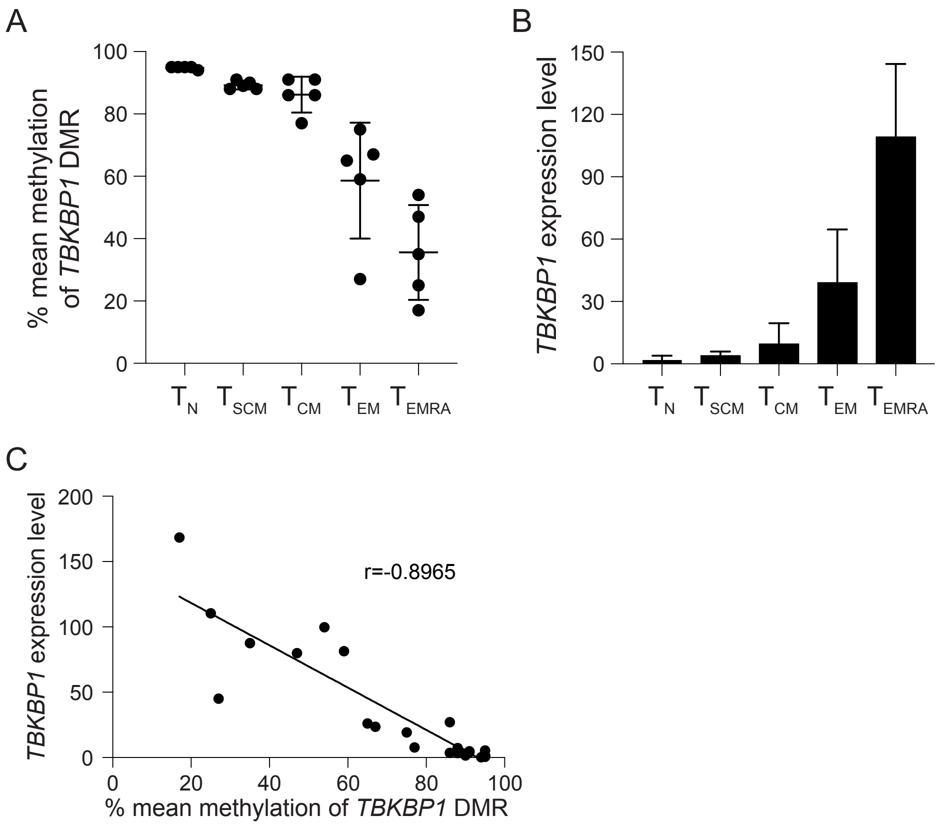

Supplementary Figure 3: Correlation between methylation level of *TBKBP1* DMR and *TBKBP1* expression in CD8⁺ T cells from CMV-seronegative donors. (A) Mean methylation level of *TBKBP1* DMR in sorted CD8⁺ T cell subpopulations T_N, T_{SCM}, T_{CM}, T_{EM}, and T_{EMRA}. Samples from five CMV-seronegative donors are shown. **(B)** *TBKBP1* expression in indicated CD8⁺ T cell subsets from five CMV-seronegative donors were analyzed by RT-PCR. **(C)** Correlation analysis visualize the relation between methylation status of *TBKBP1* DMR (x-axis) and associated *TBKBP1* gene expression (y-axis) including the linear regression line and the Pearson correlation coefficient (r).
