# Supplementary material for: DNA methylation profiling identifies TBKBP1 as potent amplifier of cytotoxic activity in CMV-specific human CD8+ T cells
Source: PLoS Pathog. 2024 Sep 26;20(9):e1012581. doi: 10.1371/journal.ppat.1012581 (PMC11460711; doi:10.1371/journal.ppat.1012581)
Supplement: S4 Fig — (A) Overview of the exon/intron structure of putative transcripts arising from the TBKBP1 gene locus on chromosome 17 (source: Ensembl GRCh38.p14). White boxes show untranslated exons, whereas black boxes represent translated exons. Ensembl transcript code, amino acid (aa) length of the protein, primer pairs for RT- PCR and the position of the TBKBP1 DMR are indicated. (B) Ratio of normalized RT-PCR signals from amplificated TBKBP1-A and TBKPB1-B using RNA of TCM, TEM and TEMRA cells. For statistical analyses, a paired two-tailed student’s t test was conducted with *, p ≤ 0.05. (PDF) [file ppat.1012581.s004.pdf]

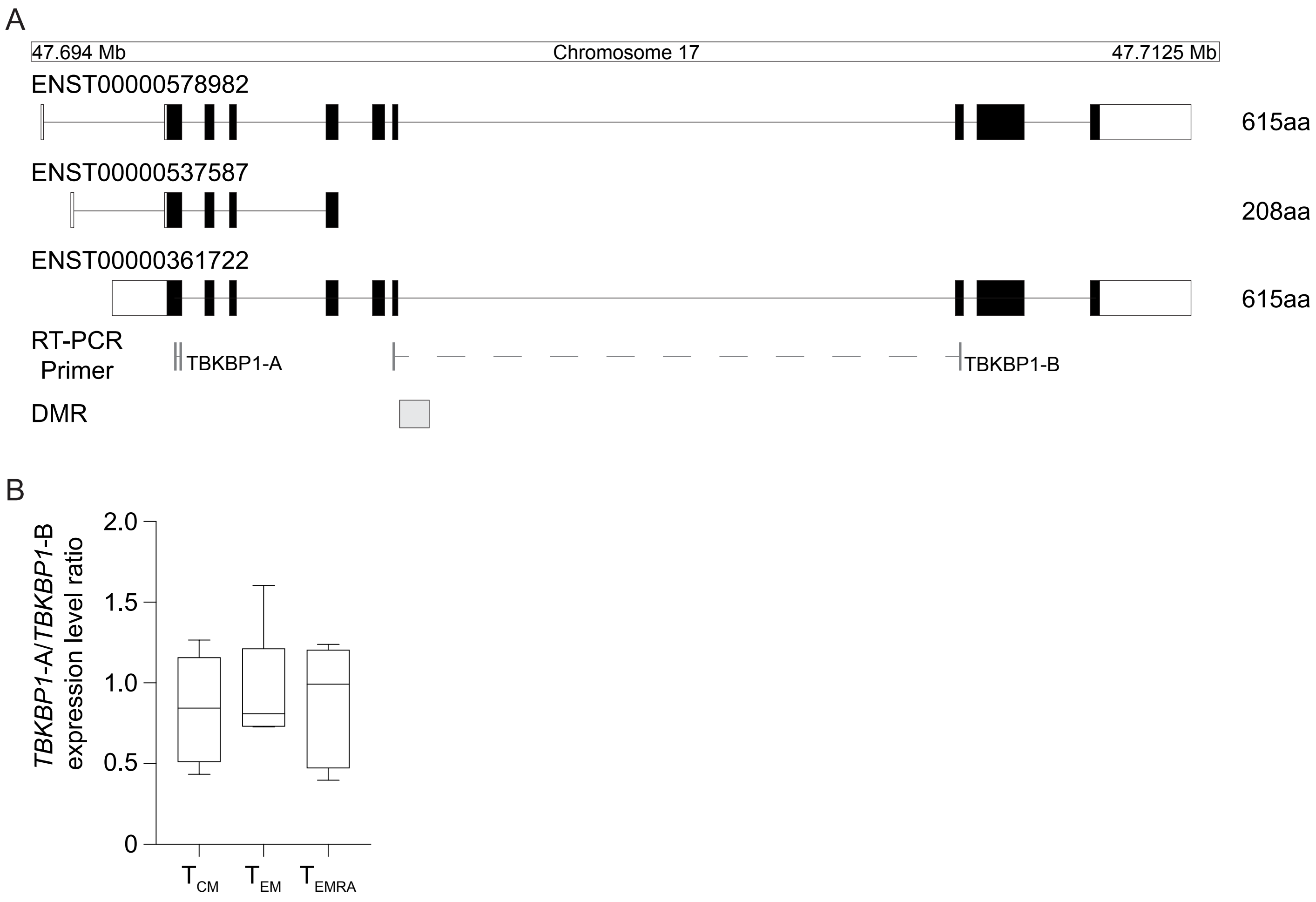


**Supplementary Figure 4: Alternative transcripts generated from the *TBKBP1* gene locus. (A)** Overview of the exon/intron structure of putative transcripts arising from the *TBKBP1* gene locus on chromosome 17 (source: Ensembl GRCh38.p14). White boxes show untranslated exons, whereas black boxes represent translated exons. Ensembl transcript code, amino acid (aa) length of the protein, primer pairs for RT-PCR and the position of the *TBKBP1* DMR are indicated. **(B)** Ratio of normalized RT-PCR signals from amplificated TBKBP1-A and TBKPB1-B using RNA of $T_{CM}$, $T_{EM}$ and $T_{EMRA}$ cells. For statistical analyses, a paired two-tailed student's t test was conducted with *, $p \leq 0.05$.
