# Supplementary material for: DNA methylation profiling identifies TBKBP1 as potent amplifier of cytotoxic activity in CMV-specific human CD8+ T cells
Source: PLoS Pathog. 2024 Sep 26;20(9):e1012581. doi: 10.1371/journal.ppat.1012581 (PMC11460711; doi:10.1371/journal.ppat.1012581)
Supplement: S5 Fig — CD8+ TN and TEMRA cells were sorted from healthy CMV-seropositive donors and cultured up to 30 days with repetitive restimulations using plate-bound anti-human CD3 and anti-human CD28 antibodies. Every 5 days, cells were harvested, washed and an aliquot was collected to determine the expression of CD8, CCR7, CD45RA, CD62L, CD95, and CD28 by flow cytometry. Representative flow cytometry plots from 5 independent cultures are depicted. (PDF) [file ppat.1012581.s005.pdf]

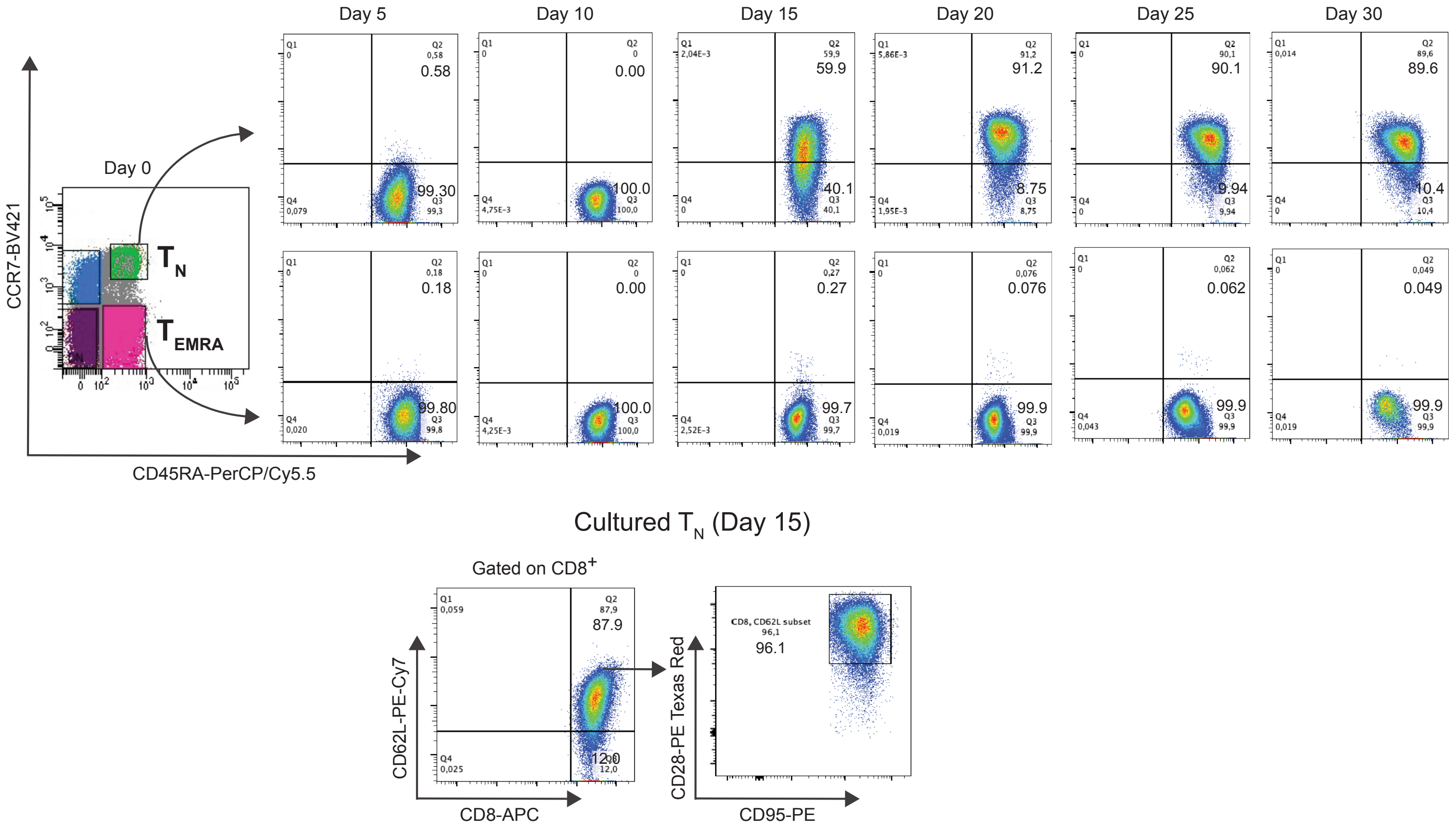

Supplementary Figure 5: Phenotypic characterisation of CD8⁺ T_N and T_{EMRA} cells during long-term cultivation. CD8⁺ T_N and T_{EMRA} cells were sorted from healthy CMV-seropositive donors and cultured up to 30 days with repetitive restimulations using plate-bound anti-human CD3 and anti-human CD28 antibodies. Every 5 days, cells were harvested, washed and an aliquot was collected to determine the expression of CD8, CCR7, CD45RA, CD62L, CD95, and CD28 by flow cytometry. Representative flow cytometry plots from 5 independent cultures are depicted.
