# Supplementary material for: DNA methylation profiling identifies TBKBP1 as potent amplifier of cytotoxic activity in CMV-specific human CD8+ T cells
Source: PLoS Pathog. 2024 Sep 26;20(9):e1012581. doi: 10.1371/journal.ppat.1012581 (PMC11460711; doi:10.1371/journal.ppat.1012581)
Supplement: S6 Fig — (A) Workflow describing the steps involved in sample processing for immunoblotting experiments. Briefly, after 48 hours of activation, PBMCs obtained from CMV-seronegative donors were transduced with pMP71-based vectors. From both empty vector (EV)- and TBKBP1-transduced samples, CD8+mCherry+ cells were sorted according to the depicted gating strategy and restimulated with anti-human CD3 and anti-human CD28 antibodies for 15 minutes followed by cross-linking with goat anti-mouse IgG, while keeping unstimulated cells as controls. Both unstimulated and restimulated cells from EV and TBKBP1 samples were subjected to immunoblotting to determine the expression levels of TBKBP1, TBK1, pTBK1, and GAPDH. (B) Representative flow cytometry plots show gating strategy (left) for the sorting of CD8+mCherry+ T cells (top: EV control; bottom: TBKBP1 overexpression) and post-sort purity (right) from one out of four donors. (C) Bar plots showing the mRNA expression of TBKBP1 in sorted CD8+mCherry+ T cells from both TBKBP1-overexpressing samples and EV-transduced controls relative to RSP9 mRNA expression. (PDF) [file ppat.1012581.s006.pdf]

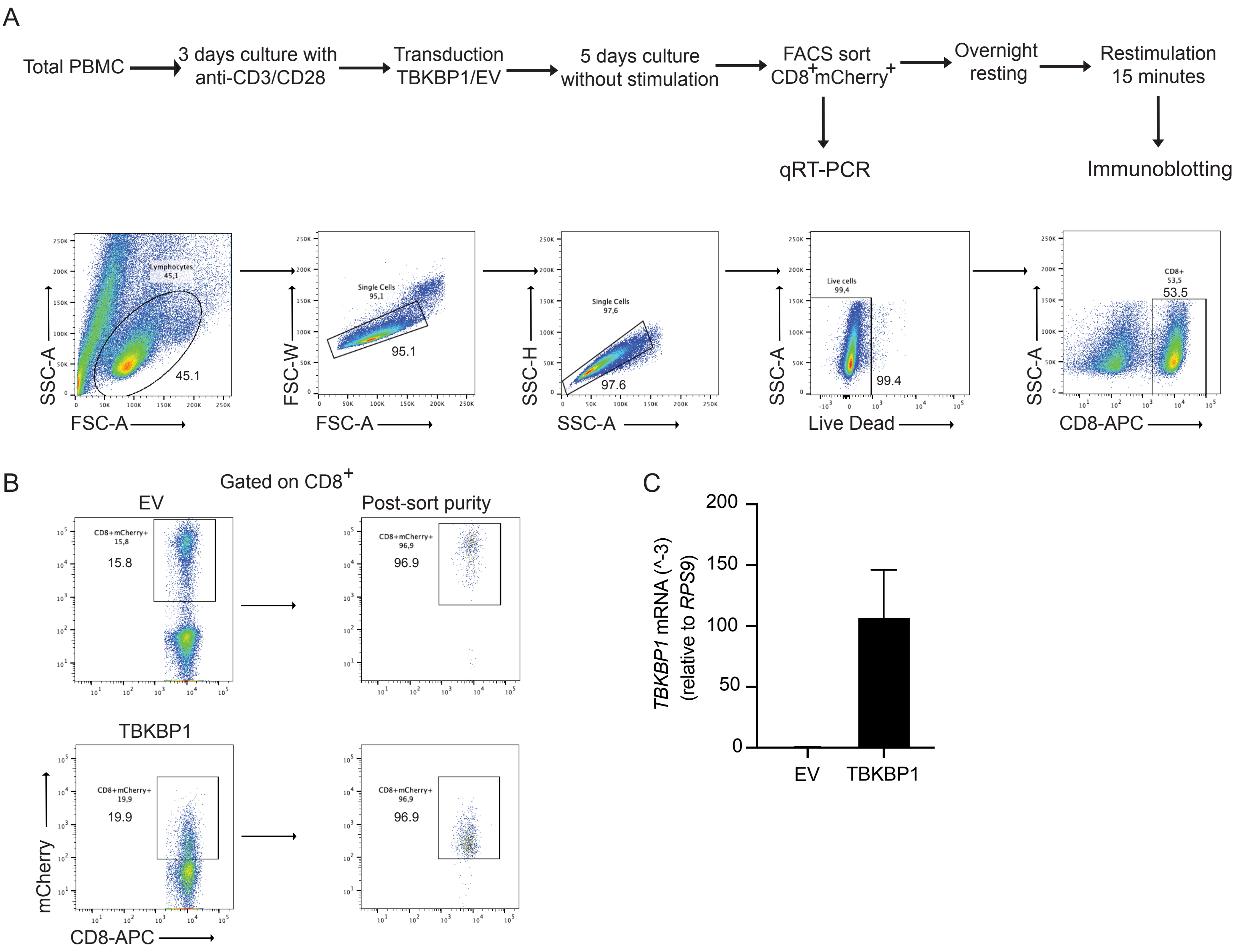

Supplementary Figure 6: Validation of retroviral TBKBP1 overexpression in CD8⁺ T cells. (A) Workflow describing the steps involved in sample processing for immunoblotting experiments. Briefly, after 48 hours of activation, PBMCs obtained from CMV-seronegative donors were transduced with pMP71-based vectors. From both empty vector (EV)- and TBKBP1-transduced samples, CD8⁺mCherry⁺ cells were sorted according to the depicted gating strategy and restimulated with anti-human CD3 and anti-human CD28 antibodies for 15 minutes followed by cross-linking with goat anti-mouse IgG, while keeping unstimulated cells as controls. Both unstimulated and restimulated cells from EV and TBKBP1 samples were subjected to immunoblotting to determine the expression levels of TBKBP1, TBK1, pTBK1, and GAPDH. **(B)** Representative flow cytometry plots show gating strategy (left) for the sorting of CD8⁺mCherry⁺ T cells (top: EV control; bottom: TBKBP1 overexpression) and post-sort purity (right) from one out four donors. **(C)** Bar plots showing the mRNA expression of *TBKBP1* in sorted CD8⁺mCherry⁺ T cells from both TBKBP1-overexpressing samples and EV-transduced controls relative to *RSP9* mRNA expression.
