# Supplementary material for: DNA methylation profiling identifies TBKBP1 as potent amplifier of cytotoxic activity in CMV-specific human CD8+ T cells
Source: PLoS Pathog. 2024 Sep 26;20(9):e1012581. doi: 10.1371/journal.ppat.1012581 (PMC11460711; doi:10.1371/journal.ppat.1012581)
Supplement: S7 Fig — TBKBP1-overexpressing CD8+ T cells and EV-transduced controls were generated, and successfully transduced CD8+mCherry+ T cells were sorted by flow cytometry, serum-starved overnight, preincubated with the PKCθ inhibitor or DMSO as control, and subsequently short-term stimulated. Samples from EV-transduced or TBKBP1-overexpressing CD8+ T cells were subjected to immunoblotting to determine the expression of TBKBP1, pTBK1, and total TBK1. The analysis of α-Tubulin expression served as loading control. Data from two independent donors are depicted. (PDF) [file ppat.1012581.s007.pdf]

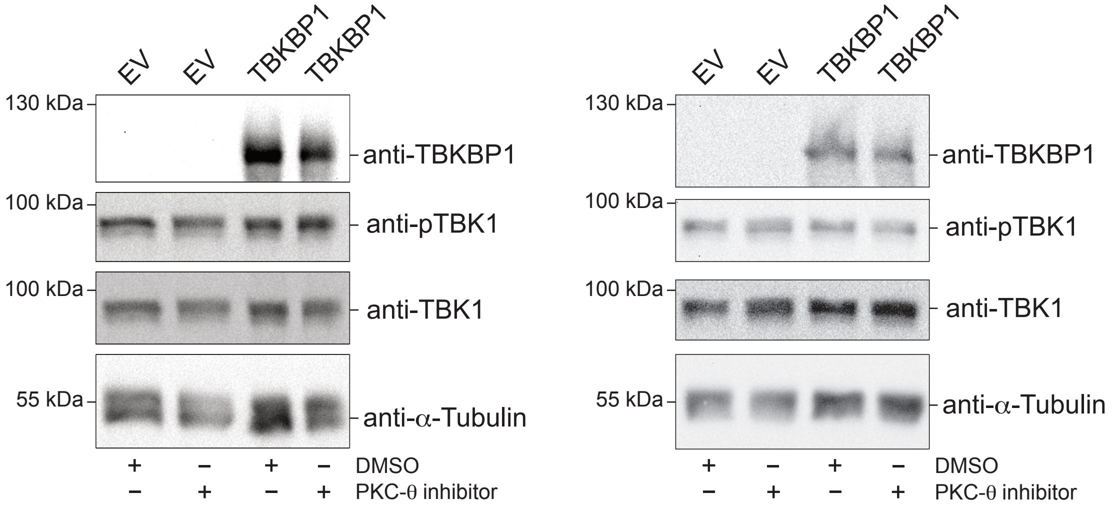

Supplementary Figure 7: Effect of PKCθ inhibition in $CD8^+$ T cells on phosphorylation of TBK1. TBKBP1-overexpressing $CD8^+$ T cells and EV-transduced controls were generated, and successfully transduced $CD8^+mCherry^+$ T cells were sorted by flow cytometry, serum-starved overnight, preincubated with the PKCθ inhibitor or DMSO as control, and subsequently short-term stimulated. Samples from EV-transduced or TBKBP1-overexpressing $CD8^+$ T cells were subjected to immunoblotting to determine the expression of TBKBP1, pTBK1, and total TBK1. The analysis of α-Tubulin expression served as loading control. Data from two independent donors are depicted.
