# Supplementary material for: DNA methylation profiling identifies TBKBP1 as potent amplifier of cytotoxic activity in CMV-specific human CD8+ T cells
Source: PLoS Pathog. 2024 Sep 26;20(9):e1012581. doi: 10.1371/journal.ppat.1012581 (PMC11460711; doi:10.1371/journal.ppat.1012581)
Supplement: S8 Fig — PBMCs isolated from healthy CMV-seronegative donors were stimulated with plate-bound anti-human CD3 and anti-human CD28 antibodies and subsequently co-transduced with mTCR and TBKBP1- or EV-mCherry plasmids. Successfully transduced CD8+mTCR+mCherry+ T cells were sorted using flow cytometry. Representative flow cytometry plots from 3 independent donors show the gating strategy for sorting of CD8+mTCR+mCherry+ T cells and post-sort purity. (PDF) [file ppat.1012581.s008.pdf]

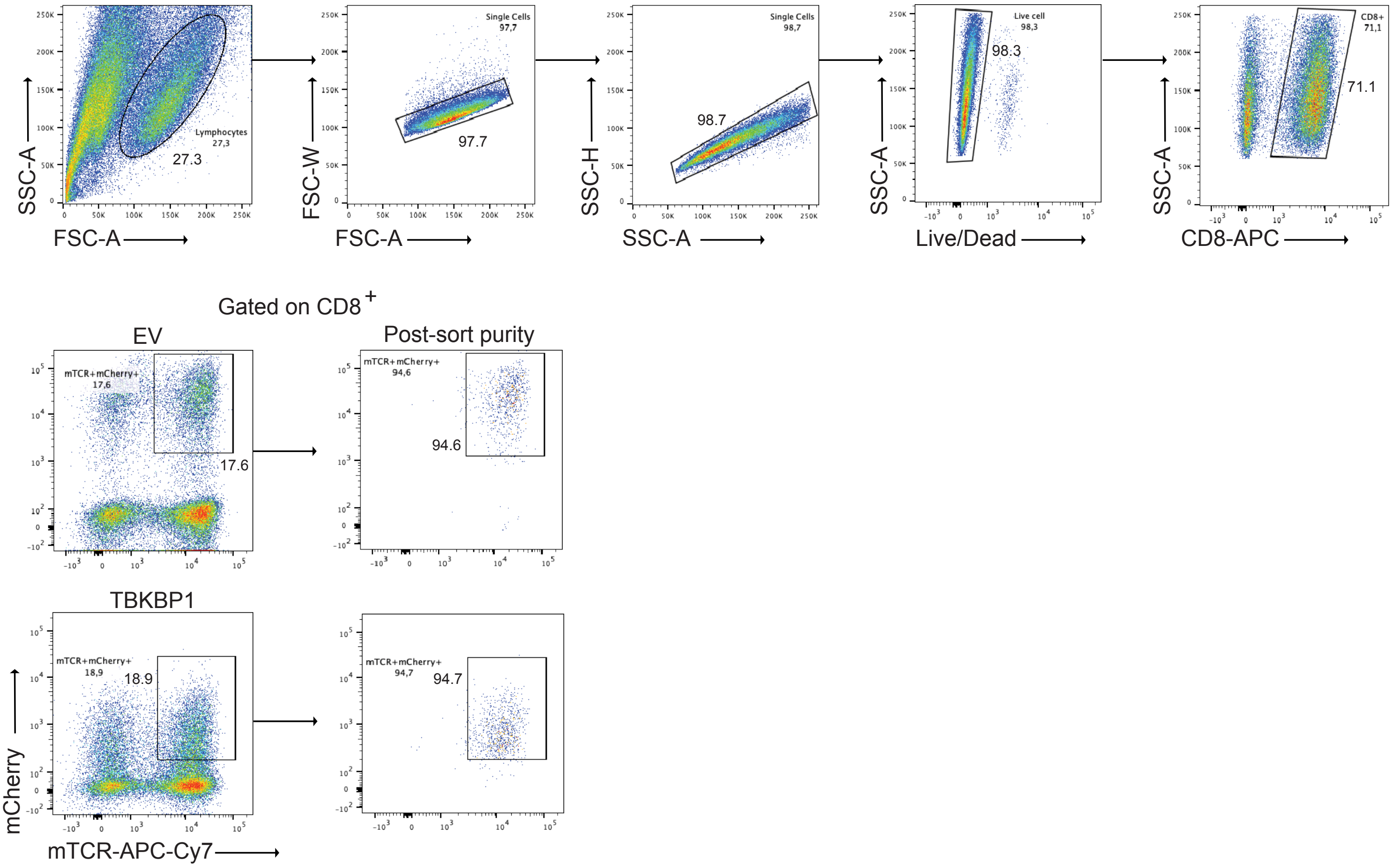

Supplementary Figure 8: Gating strategy for sorting of TBKBP1-overexpressing CD8$^+$ T cells and EV-transduced controls. PBMCs isolated from healthy CMV-seronegative donors were stimulated with plate-bound anti-human CD3 and anti-human CD28 antibodies and subsequently co-transduced with mTCR and TBKBP1- or EV-mCherry plasmids. Successfully transduced CD8$^+$mTCR$^+$mCherry$^+$ T cells were sorted using flow cytometry. Representative flow cytometry plots from 3 independent donors show the gating strategy for sorting of CD8$^+$mTCR$^+$mCherry$^+$ T cells and post-sort purity.
