# Supplementary material for: DNA methylation profiling identifies TBKBP1 as potent amplifier of cytotoxic activity in CMV-specific human CD8+ T cells
Source: PLoS Pathog. 2024 Sep 26;20(9):e1012581. doi: 10.1371/journal.ppat.1012581 (PMC11460711; doi:10.1371/journal.ppat.1012581)
Supplement: S9 Fig — PBMCs isolated from healthy CMV-seronegative donors were stimulated with plate-bound anti-human CD3 and anti-human CD28 antibodies and subsequently co-transduced with mTCR and TBKBP1- or EV-mCherry plasmids. Successfully transduced CD8+mTCR+mCherry+ T cells were sorted from both TBKBP1-overexpressing samples and EV-transduced controls using flow cytometry and co-cultured with CMV-infected MRC-5 cells followed by the ARMATA. 36 hours after infection, culture supernatants were harvested and cytokine profiles determined from cultures of CMV-infected MRC-5 cells without the addition of CD8+ T cells (w/o T cells), in the presence of EV-transduced mTCR 5–2+ CD8+ T cells (EV) or in the presence of TBKBP1-overexpressing mTCR 5–2+ CD8+ T cells (TBKBP1) with E:T ratios of 0.5:1 (left) and 1:1 (right) for indicated cytokines. Data for IFN-γ, Granzyme A, Granzyme B and Perforin from the E:T ratio of 1:1 are shown in Fig 7. Data from 3 independent experiments with 2 technical replicates each are shown. Black dots indicate frequencies from individual donors and grey bar mean values with SD. For statistical analyses, a paired two-tailed student’s t test (leaving out “w/o T cell” group) was conducted with *, p ≤ 0.05 and **, p ≤ 0.01. (PDF) [file ppat.1012581.s009.pdf]

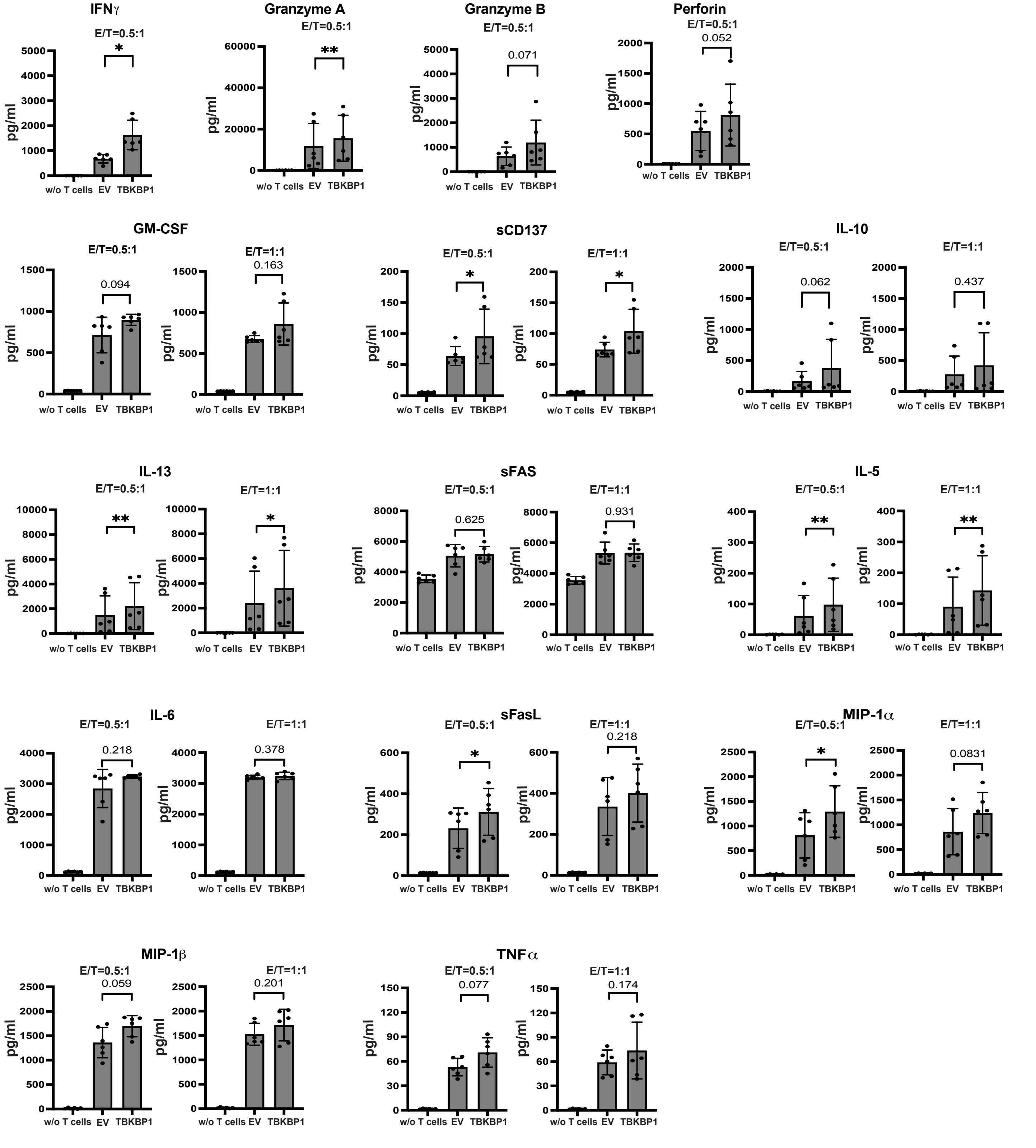

Supplementary Figure 9: Quantification of cytokines from the ARMATA.

PBMCs isolated from healthy CMV-seronegative donors were stimulated with plate-bound anti-human CD3 and anti-human CD28 antibodies and subsequently co-transduced with mTCR and TBKBP1- or EV-mCherry plasmids. Successfully transduced $CD8^+mTCR^+mCherry^+$ T cells were sorted from both TBKBP1-overexpressing samples and EV-transduced controls using flow cytometry and co-cultured with CMV-infected MRC-5 cells followed by the ARMATA. 36 hours after infection, culture supernatants were harvested and cytokine profiles determined from cultures of CMV-infected MRC-5 cells without the addition of $CD8^+$ T cells (w/o T cells), in the presence of EV-transduced mTCR $5\text{-}2^+$ $CD8^+$ T cells (EV) or in the presence of TBKBP1-overexpressing mTCR $5\text{-}2^+$ $CD8^+$ T cells (TBKBP1) with E:T ratios of 0.5:1 (left) and 1:1 (right) for indicated cytokines. Data for IFN-γ, Granzyme A, Granzyme B and Perforin from the E:T ratio of 1:1 are shown in Fig 7. Data from 3 independent experiments with 2 technical replicates each are shown. Black dots indicate frequencies from individual donors and grey bar mean values with SD. For statistical analyses, a paired two tailed student's t test (leaving out "w/o T cell" group) was conducted with *, $p \leq 0.05$ and **, $p \leq 0.01$.
