# Supplementary material for: DNA methylation profiling identifies TBKBP1 as potent amplifier of cytotoxic activity in CMV-specific human CD8+ T cells
Source: PLoS Pathog. 2024 Sep 26;20(9):e1012581. doi: 10.1371/journal.ppat.1012581 (PMC11460711; doi:10.1371/journal.ppat.1012581)
Supplement: Attachment — Submitted filename: Yu_PPATHOGENS-D-24-00297_rebuttal_letter.docx [file ppat.1012581.s011.docx]

**Rebuttal letter for PPATHOGENS-D-24-00297**

"DNA methylation profiling identifies TBKBP1 as potent amplifier of cytotoxic activity in CMV-specific human CD8^+^ T cells"

Dear Reviewers,

We highly appreciate the interest expressed in our manuscript and would sincerely like to thank you for your constructive critique and highly useful comments, which have helped us to improve the overall quality of our manuscript.

Based on your valuable suggestions, we have created novel experimental data and integrated them into the manuscript. Major modifications comprise the analysis of TBKBP1 and TBK1 expression as well as TBK1 phosphorylation in *ex vivo* isolated T cell subsets, the analysis of the methylation status of the *TBKBP1* DMR and TBKBP1 mRNA expression in T cell subsets from CMV-seronegative donors, the analysis of alternative splicing of *TBKBP1* transcripts in T cell subsets, and experiments studying the impact of PKCθ inhibition on TBK1 phosphorylation and the functional properties of in TBKBP1-overexpressing T cells.

In total, we have added one new main (Figure 5) and three new Supplementary Figures (S3, S4 & S7), added one additional panel to the main Figure 6 (Figure 6E), modified panels A&C in main Figure 7, and modified Supplementary Figures S2&S9. For clarity, we have split former Figure 3 into two Figures (now Figure 3 & 4). Furthermore, we provide one additional Figure for the Reviewers. In the revised version of the manuscript, all changes are highlighted in red.

While our paper was under review, we published a paper in which the virus reduction assay was described in detail and named *assay for rapid measurement of antiviral T cell activity*, abbreviated ARMATA. As we use this assay in the present manuscript, we cited the paper (Khan et al., 2024) and changed "virus reduction assay" into ARMATA to avoid any misunderstanding.

Please find below our detailed point-by-point responses to your comments. We believe that we have addressed all concerns and hope that our revised manuscript is now worth getting published in PLoS Pathogens.

Yours sincerely,

Jochen Huehn

**Point-by-point response to reviewer’s questions of submission PPATHOGENS-D-24-00297**

**I. Major Issues: Key Experiments Required for Acceptance**

***Reviewer #1***

**Major Issue 1:** Key missing piece of data: enhanced phosphorylation of TBK1 in the TEMRA cells or CMV-specific TEMRA in the absence of TBKBP1 overexpression. Overexpression experiments can lead to non-physiological outcomes and it remains possible that only artificially very high levels of TBKBP1 have an effect on TBK1 and T cell function. If so, the demethylation of TBKBP1 may not lead to any change in T cell function, but have some other effects.

**Our response to Major Issue 1:** Reviewer 1 points to an important effect of protein overexpression. We fully agree that artificially increased expression of a signalling pathway protein might not only influence one type of signalling, but may also have unspecific side-effects by targeting other mediators. Thus, we followed the reviewers’ recommendation and assessed the expression of TBKBP1 and TBK1, as well as the phosphorylation of TBK1 in *ex vivo* isolated, unmanipulated T_N_, T_CM_, T_EM_, and T_EMRA_ cells from CMV-seropositive donors by immunoblot. We could demonstrate that TBKBP1 was barely expressed in T_N_ cells, but clearly detectable in T_CM_, T_EM_ and T_EMRA_ cells. Similar findings were observed for the expression levels of total and phosphorylated TBK1. A quantification of band intensities revealed a significantly increased pTBK1/TBK1 ratio in T_EMRA_ cells when compared to T_CM_ cells, while T_EM_ cells only showed a trend for an increased pTBK1/TBK1 ratio (p=0.062). Whether TBKBP1 is directly and solely involved in phosphorylation of TBK1 or is participating also in other signaling pathways cannot be answered by this approach. A detailed T cell signalling pathway study would be required for clarification of all TBKBP1 functions.

Our new findings, which were bundled in the **new Figure 5** and mentioned at various sections of the manuscript (**see lines 75-76, 152-153, 290-300, 390-391, 660-662, 676-685**), provide evidence that the TBK1 signaling pathway is more active in terminally differentiated effector memory CD8^+^ T cells. Furthermore, we mentioned the limitations of overexpression systems in the Discussion section (**see lines 407-410**).

**Major Issue 2:** Additionally, the relative amount of TBKBP1 and TBK1 in the overexpression system vs the unmanipulated TEMRA cells should be shown. Is the expression too low to detect by Western blot without overexpression?

**Our response to Major Issue 2:** We apologize that this type of information was missing in the first version of the manuscript. As mentioned in our response to Major Issue 1, for the revised manuscript we analyzed the expression of TBKBP1 and TBK1, as well as the phosphorylation of TBK1 in *ex vivo* isolated CD8^+^ T cell subsets isolated from five healthy donors by immunoblot. TBKBP1 expression levels were detectable in all memory-type T cell subsets, but too low to allow for quantification of band intensities. In contrast, TBK1 expression was clearly detectable in all memory-type T cell subsets and could be quantified. Here, the overall protein levels were similar and did not show any significant differences between the subsets (**see new Figure 5A&B**). Together with the findings from the overexpression system, we conclude that an increase in *TBKBP1* expression in T_EMRA_ cells is not accompanied by a significant increase in TBK1 expression.

**Major Issue 3:** Ideally, the investigators should also show that knockdown of TBKBP1 in TEMRA cells would reduce pTBK1 and T cell efficacy. Even if levels of TBKBP1 are too low to detect by Western blot, a change in function should be evident if their conclusions are not an artifact of overexpression.

**Our response to Major Issue 3:** We fully agree with the reviewer that assessing the impact of *TBKBP1* knockdown in T_EMRA_ cells on the phosphorylation of TBK1 and on the functional properties of the cells would be highly informative. Therefore, we followed this recommendation and performed CRISPR/Cas9-mediated *TBKBP1* knockout experiments using combined T_EM_ and T_EMRA_ cells, which both showed increased *TBKBP1* expression levels, from CMV-seropositive donors. The experiments were challenging, and although we tested the material from six donors in technical duplicates, we were unable to properly analyze the samples for various reasons. First, the knockout efficacy was only moderate and RNP-based targeting approach did not allow for sorting of or gating on successfully transfected cells. Secondly, the nucleofection procedure resulted in an increased cell death of the primary T cells, which prevented us from analyzing the samples by immunoblot in parallel to RT-PCR and flow cytometry. Indeed, most of the immunoblots did not meet our quality criteria. Yet, we were able to perform a series of RT-PCR- and flow cytometry-based assays, in which we could demonstrate that a reduction of *TBKBP1* expression is accompanied by a reduced IFN‑γ expression upon restimulation. This phenotype was observed in samples from three out of four donors, in which a reduction of *TBKBP1* expression by >30% could be reached (**see Reviewer Figure 1A**). For the samples of a single donor (#3), we were able to successfully generate immunoblots with sufficient quality. Here, we could show that a reduction of *TBKBP1* expression results in a reduced pTBK1/TBK1 ratio after restimulation (**see Reviewer Figure 1B, 1C**). We apologize for not being able to provide data of a sufficient number of independent experiments that can be included in the revised manuscript. Yet, we hope that the reviewer is convinced that our conclusions from the TBKBP1 overexpression experiments are not an artifact of overexpression, but could be confirmed by *TBKBP1* knockout experiments.

**Major Issue 4:** For the methylation patterns, CMV-specific TEMRA were compared to bulk memory and naïve CD8 T cells. In general, this is reasonable but it leaves out some information. It wasn’t clear to me whether the results were specific to TEMRA or CMV-specific T cells or CMV-specific TEMRA. Are the same patterns true the TBKBP1 levels and methylation are compared to CMV-specific Tmem, or bulk TEMRA cells regardless of specificity or TEMRA cells in CMV-negative donors? I know there are smaller numbers of CMV-specific Tmem and TEMRA cells in CMV-negative donors, but there should be enough for qPCR of TBKBP1 levels at least, if not some analyses of methylation patterns.

**Our response to Major Issue 4:** We have to apologize for not having this explained more clearly. We had simply missed to mention the CMV-seropositive status of the donors in this part of the manuscript. In the revised version of the manuscript, we have now added the information about the CMV status of the donors (**see lines 237-238**). Furthermore, we have extended this part of the result section by providing corresponding data from CD8^+^ T cell subsets isolated from five CMV-seronegative donors (**see new S3 Fig**). Also here, an inverse correlation of *TBKBP1* DMR methylation and mRNA expression was observed, indicating that the DMR‑mediated transcriptional control of the *TBKBP1* gene locus is independent of the CMV status. Our new findings, which were mentioned at various sections of the manuscript (**see lines 249-254, 391-398, 478-480**), suggest that the observed epigenetic regulation of *TBKBP1* expression could be also caused by immune responses to other pathogens.

***Reviewer #2:***

Nil

***Reviewer #3:***

**Major Issue 5:** Fig. 3 – all data here correlates DNA methylation levels with mRNA/transcripts levels. Transcript levels are determined by qPCR with primers spanning exons 1-2, if correct (although please see note in Minor issues regarding primers also). However, the DMR is further down the gene beyond further intron/exon breaks. DNA methylation is also known to govern splicing events, which is also linked to differentiation of cells and changing transcript profiles. Therefore, the authors must show the total profile of possible transcripts from this gene (i.e. regular PCR minimally or RNA-seq) across their differentiating cell populations and also show protein expression levels of TBKBP1, which of course is the presumed effector of their final phenotype.

**Our response to Major Issue 5:** We are very grateful to this reviewer for pointing out to this important aspect regarding the epigenetic control of splicing events. We followed the reviewers’ recommendation and took a closer look at the *TBKBP1* transcripts. In the revised version of the manuscript, we now show the putative protein-coding *TBKBP1* transcripts obtained from the Ensembl data base (**see new S4A Fig**). Translation of transcripts ENST00000361722 and ENST00000578982 lead to the same polypeptide chain of 615 amino acids. ENST00000537587 might result in the production of a shorter protein with 208 amino acids. To analyze if changes in the methylation status in the CD8^+^ T cell subsets led to alternative splicing events and generated different transcripts, we here applied two RT-PCR reactions with primer pairs either binding to the first translated exon (TBKBP1-A) or to the sixth and seventh translated exon (TBKBP1-B) (**see new S4A Fig**). We extracted total RNA from *ex vivo* isolated T_CM_, T_EM_ and T_EMRA_ cells, which display distinct *TBKBP1* DMR methylation levels, performed the two RT-PCR reactions and determined the ratio of the two *TBKBP1* qRT-PCR transcripts. As shown in the **new S4B Fig**, the statistical analysis of the results did not reveal any significant difference in the ratio between the three memory-type CD8^+^ T cell subsets, confirming that a change in the methylation status of the *TBKBP1* DMR does not lead to altered transcriptional profiles. This finding, which was mentioned at various sections of the manuscript (**see lines 76, 154-156, 233, 255-265, 607-615**), was in line with results from a recently published study that did not detect TBKBP1 protein variants in total CD4^+^ and CD8^+^ T cells as well as NKT cells (Zhu et al., 2018 – reference #64)

**Major Issue 6:** Fig. 4 – since the authors hypothesize in Fig 4A that PKCθ is the kinase responsible for phosphorylation of TBK1 during their experiment, to test this fully they must test this through use of a highly selective PKCθ inhibitor (e.g. HY-112681). Moreover, immunoprecipitation experiments would also be useful to show that TBKBP1/TBK1 and/or TBKBP1/CARD10 are interacting under the conditions of their experiment to prove direct effects.

**Our response to Major Issue 6:** We thank this reviewer for raising this point. Based on studies in other cell types, we had hypothesized that PKCθ might play a role in the phosphorylation of TBK1 in CD8^+^ T cells (Fig. 4A in initially submitted manuscript). To support this hypothesis, we performed experiments with the highly selective PKCθ inhibitor HY-112681 as recommended by the reviewer. Since the inhibitor showed a marked toxic effect in initial experiments, especially under longer cell culture durations, we could not perform a killing assay to study the impact of the enzymatic activity of PKCθ on the cytotoxic activity of the CD8^+^ T cells. Furthermore, in the ARMATA, it would have been impossible to discriminate between effects of the inhibitor on the CD8^+^ T cells or the infected MRC‑5 cells. Thus, we decided to assess the impact of the PKCθ inhibitor HY‑112681 on the phosphorylation of TBK1 as well as the effector cytokine-producing capacity using TBKBP1-overexpressing CD8^+^ T cells. Notably, immunoblot analyses revealed no effect of the PKCθ inhibitor on the phosphorylation of TBK1, neither in TBKBP1-overexpressing nor EV-transduced control cells (**see new S7 Fig**). However, we observed an effect of the PKCθ inhibitor on the IFN‑γ expression levels in TBKBP1-overexpressing CD8^+^ T cells, while the inhibitor had no effect in EV-transduced control cells (**see new Figure 6E**). Taken together, our new findings suggest that PKCθ is involved in the TBK1 signalling pathway, but independently of TBK1 phosphorylation. These new findings were mentioned in various sections of the manuscript (**see lines 315-333, 448-449, 475-476, 514-516, 669-670, 702-714**). Furthermore, since we could not support the hypothesis that PKCθ plays a role in the phosphorylation of TBK1 in CD8^+^ T cells, we deleted the scheme from the previous Figure 4A and also toned down the wording in the corresponding sections of the manuscript.

Although we agree with this reviewer that immunoprecipitation experiments would be useful to demonstrate interactions between TBKBP1 and TBK1 or TBKBP1 and CARD10 under the conditions of our experiments, we refrained from doing these experiments since in our opinion they go beyond the scope of this manuscript.

**Major Issue 7:** Fig. 5 – the data here are compelling, however further inhibitor use during experiments should help to confirm that the pathway the authors believe is activated is the actual effector, i.e. use of highly selective PKCθ (e.g. HY-112681) and/or TBK1 (e.g. GSK8612) inhibitors, which should eliminate the difference in killing between EV and TBKBP1 over-expressing cells. This type of experiment presumably could also be conducted with CD8-pos T cells from sero-positive donors and CMV infected target cells to show a decrease in the natural ability of these cells to kill.

**Our response to Major Issue 7:** We thank the reviewer for this suggestion. As mentioned in our detailed response to Major Issue 6, we could not use inhibitors for PKCθ or TBK1 in the ARMATA to study the impact of these enzymes on the cytotoxic activity of the CD8^+^ T cells since initial experiments showed a marked toxic effect especially under longer cell culture durations and it would have been impossible to discriminate between effects of the inhibitors on the CD8^+^ T cells or the infected MRC‑5 cells. Thus, we deleted the scheme from the previous Figure 4A and also toned down the wording in the corresponding sections of the manuscript.

**Minor Issues: Editorial and Data Presentation Modifications**

***Reviewer #1***

**Minor Issue 1:** While the trends are obvious in Figure 5C, the differences appear to be very subtle and the power of the analyses appears weak with the possible exception of IFNg expression. I believe that the samples derive from individual donors transfected with either the EV or the TBKBP1. It might help if the paired samples were indicated in the figure as in 5B.

**Our response to Minor Issue 1:** We apologize for the lack of clarity in the presentation of the data in **Fig 7** (previous Fig 5). In **Fig 7C**, we have now used different symbols to make it easier to identify the data corresponding to samples from the same donor.

***Reviewer #2***

**Minor Issue 2:** One point is that the CMV-specific T cells are (1) recently activated and (2) EMRA phenotype. These are compared against CD27^+^CD28^+^ early differentiated effector T cells without recent stimulation. Whilst later experiments show that TBKTB1 methylation is not altered by cell proliferation, and pyrosequencing of EM vs EMRA is undertaken, I would mention this limitation in the Discussion section.

**Our response to Minor Issue 2:** We apologize that our initial description of the sorted T cell subsets in the first version of the manuscript was not clear enough. Therefore, as recommended by the reviewer we have added information about the phenotype of the Tmem population to the Discussion section (**see lines 372-374**) and removed the term ‘bulk’ from Abstract, Results and Material & Methods.

**Minor Issue 3:** The work of the Peterson group in this area should be mentioned and discussed - e.g. PMID: 38370415; PMID: 35397197.

**Our response to Minor Issue 3:** We thank the reviewer for this valuable advice. We have cited Turk et al. from the Peterson group (reference #79) and discussed their interesting findings regarding the epigenetic regulation of *ADAM28* (**see lines 423-428**).

**Minor Issue 4:** There is also some work on genetic variants of TBKBP1 with HHV7 infection (PMID: 33109261)

**Our response to Minor Issue 4:** We thank the reviewer for drawing our attention to this study. We are aware of this paper, where a position 5.29 KB upstream of the *TBKBP1* gene locus was associated with HHV7 infection. Yet, since this genetic variant is located far away from the identified *TBKBP1* DMR outside of the main gene body, and it is unclear whether it might rather be associated with the nearby gene *TBX21*, we considered the association as too speculative and thus have decided to not cite this reference in the discussion.

***Reviewer #3***

**Minor Issue 5:** Fig. 2 – do all the genes shown with DMRs here have changes to transcript level correlating with DNA methylation level consistent with TBKBP1 data, or is this restricted to TBKBP1? Is qPCR data available for other genes as per Fig. 3B/C. This of course would determine the importance of DMR profiling across T cell populations, since only one gene has been focused up beyond this point.

**Our response to Minor Issue 5:** We thank the reviewer for raising this point. Unfortunately, qPCR data are not available for the other genes depicted in Fig 2. In the present study, we have selected *TBKBP1* for further investigations, yet we agree with the reviewer that studying the expression of the other DMR-linked genes in T cell subsets might result in interesting findings in future studies. Here, we have cited additional references (#47 & 48) to better describe the known roles of *KLRD1*, *TBX21*, *ZEB2*, and *S1PR5* in CD8^+^ T cell subsets.

**Minor Issue 6:** Fig. 5A – brightfield images would be useful to show the absence of further cells. Although the T cells are presumably much smaller, can their presence be shown with the red/mCherry channel here?

**Our response to Minor Issue 6:** We thank the reviewer for this suggestion. We have added brightfield images to the corresponding fluorescent images in **Fig 7A** (previous Fig 5A).

**Minor Issue 7:** Discussion - Is there any known correlation between TBKBP1 mutations and natural susceptibility to virus/herpesvirus infections?

**Our response to Minor Issue 7:** As mentioned by reviewer #2 (see Minor Issue 4), a recent study by Kachuri et al. published in Genome Medicine has identified a genetic variant of *TBKBP1* being associated with HHV7 infection (PMID 33109261). Yet, as outlined in more detail in our response to this Minor Issue, the association of this variant with the *TBKBP1* locus has not been validated, wherefore we decided to not cite this paper.

**Minor Issue 8:** Discussion - Reduced DNA methylation at the DMR in TBKBP1 is clear during differentiation, however some discussion must be made regarding why this site in particular is important – further areas in the same intron appear also de-methylated vs the two other cell populations.

**Our response to Minor Issue 8:** We thank the reviewer for this comment. Indeed, it seems that further regions in the same intron as the *TBKBP1* DMR appear to be demethylated in T(CMV) when compared to Tmem or T_N_ cells, however only the *TBKBP1* DMR was identified as differentially methylated region when the pipeline was applied. To address the concern of the reviewer, we have discussed that this *TBKBP1* DMR might be important through the co-operation of bound transcription factors with the known TBKBP1 enhancer region located between exon 4 and 5 (**see lines 382-385**).

**Minor Issue 9:** M&Ms - The primers used for qPCR have a number of mismatches with the available human TBKBP1 gene sequence available from NCBI. Is there a particular reason for this?

**Our response to Minor Issue 9:** We apologize for this mistake and thank the reviewer for spotting it. The TBKBP1 overexpression cassette contains a codon-optimized cDNA. Therefore, detection of the transcript requires primers that are not 100% identical to the reference sequence. We have included the correct information about the primers in the revised manuscript (**see lines 607-611**).

**Minor Issue 10:** Supplement - S2 Fig – cell type is denoted by box color here – does this mean that all black boxes are TN (naïve T cells)?

**Our response to Minor Issue 10:** We apologize for the unclear labelling of sort gates. We have changed the color code in **S2 Fig** and revised the figure legend to unequivocally identify the T_N_ cells.

**Minor Issue 11:** Supplement - Fig S6 – unlike killing of target cells, which becomes more statistically significant with an E:T ratio of 1 (vs 0.5; Fig 5B), statistical significance of changed expression of cytokines here appears generally to decrease with a greater E:T. Can this be explained?

**Our response to Minor Issue 11:** We have carefully reviewed the cytokine profiles from the ARMATA and confirmed the results. Unfortunately, we do not have an explanation for this phenomenon.

**Minor Issue 12:** Supplement - S6 Fig. legend – it must be mentioned that some data presented here is also already presented in Fig. 5.

**Our response to Minor Issue 12:** We thank the reviewer for pointing to this mistake and apologize that some bar graphs were presented twice. We decided to delete the graphs already depicted in **Fig 7C** (previous Fig 5C) from **S9 Fig** (previous S6 Fig).
